# Dynamic O-GlcNAcylation and phosphorylation attract and expel proteins from RNA polymerase II to regulate mRNA maturation

**DOI:** 10.1186/s12929-025-01135-9

**Published:** 2025-04-04

**Authors:** Aishwarya Gondane, Harri M. Itkonen

**Affiliations:** Department of Biochemistry and Developmental Biology, Faculty of Medicine, University of Helsinki, 00014 Helsinki, Finland

**Keywords:** SLAM-seq, Cyclin-dependent kinase, Nascent transcription, mRNA maturation, Intronic poly-adenylation, O-GlcNAc transferase

## Abstract

**Background:**

Phosphorylation and O-GlcNAcylation are the key modifications regulating RNA Polymerase II (RNA Pol II)-driven transcription. Transcriptional kinases, cyclin-dependent kinase 7 (CDK7), CDK9 and CDK12 phosphorylate RNA Pol II, whereas O-GlcNAcylation is added by O-GlcNAc transferase (OGT) and removed by O-GlcNAcase (OGA). Currently, no study has systematically evaluated how inhibiting each of these enzyme activities impacts the assembly of the appropriate protein complexes on the polymerase and the maturation of mRNA.

**Methods:**

Here, we systematically evaluate remodeling of RNA Pol II interactome and effects on the nascent mRNA maturation by using mass spectrometry and SLAM-seq, respectively. For validation, we rely predominantly on analysis of intronic polyadenylation (IPA) sites, mitochondrial flux assays (Seahorse), western blotting and patient data.

**Results:**

We show that OGT / OGA inhibition reciprocally affect protein recruitment to RNA Pol II, and appropriate O-GlcNAcylation levels are required for optimal function of the RNA Pol II complex. These paradoxical effects are explained through IPA, because despite being prematurely poly-adenylated, these mRNAs are scored as mature in SLAM-seq. Unlike previously proposed, we show that, similar to inhibition of CDK12, also targeting CDK9 stimulates transcription of short genes at the cost of long genes. However, our systematic proteomic- and IPA-analysis revealed that these effects are mediated by distinct molecular mechanisms: CDK9 inhibition leads to a failure of recruiting Integrator complex to RNA Pol II, and we then show that depletion of Integrator subunits phenocopy the gene length-dependent effects. In contrast, CDK12 inhibition triggers IPA. Finally, we show that dynamic O-GlcNAcylation predominantly interplays with CDK9: OGT inhibition augments CDK9 inhibitor effects on mRNA maturation due to defects in transcription elongation, while OGA inhibition rescues mRNA maturation failure caused by targeting CDK9, but induces IPA.

**Conclusion:**

We show that dynamic O-GlcNAcylation is a negative regulator of mRNA biosynthesis and propose that the addition and removal of the modification serve as quality control-steps to ascertain successful generation of mature mRNAs. Our work identifies unprecedented redundancy in the regulation of RNA Pol II, which increases resilience towards transcriptional stress, and also underscores the difficulty of targeting transcription to control cancer.

**Graphical Abstract:**

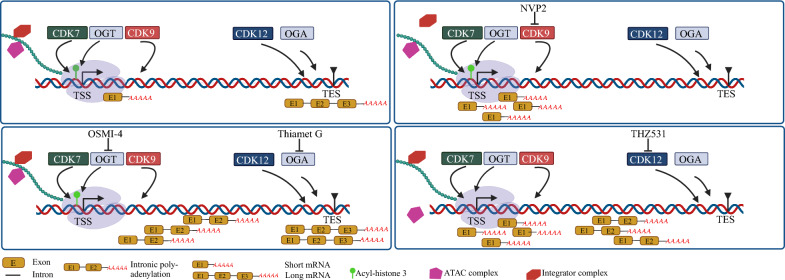

**Supplementary Information:**

The online version contains supplementary material available at 10.1186/s12929-025-01135-9.

## Introduction

Transcription is controlled by phosphorylation of the C-terminal domain (CTD) of RNA polymerase II (RNA Pol II) [1]. Cyclin-dependent kinases (CDK) 7, 9 and 12/13 phosphorylate the CTD to promote transcription initiation, to release the polymerase from promoter-proximal pausing, and to sustain the productive elongation on the long genes, respectively [2–5]. The CTD has 52 repeating units of seven amino acids; this domain generates a complex pattern, which is critical for the timely attraction of the appropriate protein machinery onto the elongating RNA Pol II to process the emerging mRNA. Kinase-dependent phosphorylation of the CTD has been established as a critical modification to regulate transcription.

RNA Pol II is also glycosylated by O-GlcNAc transferase (OGT) and this modification has been proposed to function mutually exclusive with phosphorylation [6–9]; however, the functional importance of the CTD glycosylation has not been established. *OGT* is an essential gene in human and the enzyme serves as a sensor of metabolic homeostasis because the necessary co-substrate levels fluctuate according to nutrient-availability [10–12]. In contrast to multiple CTD kinases, OGT is a unique and poorly characterized regulator of RNA Pol II activity. Notably, OGT and CTD kinases have similar K_m_-values for the CTD, which demonstrates that neither has kinetic advantage in the steady-state conditions [13]. OGT recognizes its substrates largely through interactions between the tetratricopeptide (TPR)-domain far from the active site, and, accordingly, efficient glycosylation of the CTD requires TPR-domain of OGT and a minimum of 10 CTD repeats of RNA Pol II [13, 14]. Experiments with partially purified components of the transcription machinery suggest that OGT regulates RNA Pol II promoter loading and is important at least for the expression of cell-type specific genes [7, 8]. The O-GlcNAc modification is removed by O-GlcNAcase (OGA), which is known to interact with components of the transcription elongation machinery [15]. Being the sole eraser of O-GlcNAcylation, OGA must remove the modification from RNA Pol II as well. OGT and OGA are positioned as central regulators of transcription, but their actual roles remain enigmatic.

The central role of dynamic phosphorylation and O-GlcNAcylation in transcription has motivated development of specific inhibitors as candidate therapies. OGA inhibitor Thiamet G was developed based on bioisosteric properties of an earlier generation inhibitors to increase potency and chemical stability [16]. We have participated in the development of the currently best small molecule inhibitor targeting OGT, OSMI-4 [11, 17]. Recently developed, highly specific inhibitors against the transcriptional kinases CDK7 (YKL-5-124), CDK9 (NVP2) and CDK12/13 (THZ531) [2, 4, 18], make it possible to pinpoint how each of these enzymes regulate RNA Pol II interactome and activity.

Transcription can be tracked by measuring stable RNAs (RNA-seq), but only by measuring the newly synthesized mature mRNAs, it is possible to detect direct effects on RNA Pol II-dependent transcription [19]. All the techniques to measure nascent transcription answer specific questions but come with their limitations. PRO- and GRO-seq can measure RNA Pol II activity in a very precise manner but require isolation of nuclei, which may result in the loss of transcriptional regulators and these two tools also fail to measure mRNA maturation [20]. 4sUDRB-seq relies on labeling transcripts and purification of the newly made mRNA with affinity matrix, which might result in the loss of signal of the lowly expressed genes, and again, the tool does not actually detect mRNA maturation [21]. SLAM-seq (thiol(SH)-linked alkylation for metabolic sequencing of RNA) relies on labeling of the on-going transcription with nucleotide analog, which is chemically converted rendering it recognizable as an SNP [22, 23]. SLAM-seq relies on 3’ end sequencing of poly-adenylated products, in essence, only the mature mRNAs are detected. However, SLAM-seq can in principle also identify pre-maturely poly-adenylated mRNAs. Major limitation of this tool is the labeling time, which must be empirically defined to assure robust labeling and to still detect direct effects on RNA Pol II output. We believe that SLAM-seq is the ideal tool to probe how each of the major transcriptional regulators directly contribute to mRNA maturation.

In this project, we set out to understand how dynamic O-GlcNAcylation regulates RNA Pol II interactome and activity in conjunction with the major transcriptional kinases. We show that both of the enzymes that add and remove O-GlcNAcylation, OGT and OGA, respectively, are negative regulators of mRNA maturation. However, inhibitors targeting either have only modest effects on transcription, and O-GlcNAcylation therefore serves as a non-essential regulator of transcription, which becomes essential when transcriptional kinases are inhibited. Finally, our comprehensive proteomics data along with validation experiments identify actionable combinatorial and synthetic lethal targets together with defects in the major transcriptional kinases.

## Results

### High OGT activity prevents recruitment of proteins to RNA Pol II

**Fig. 1:**
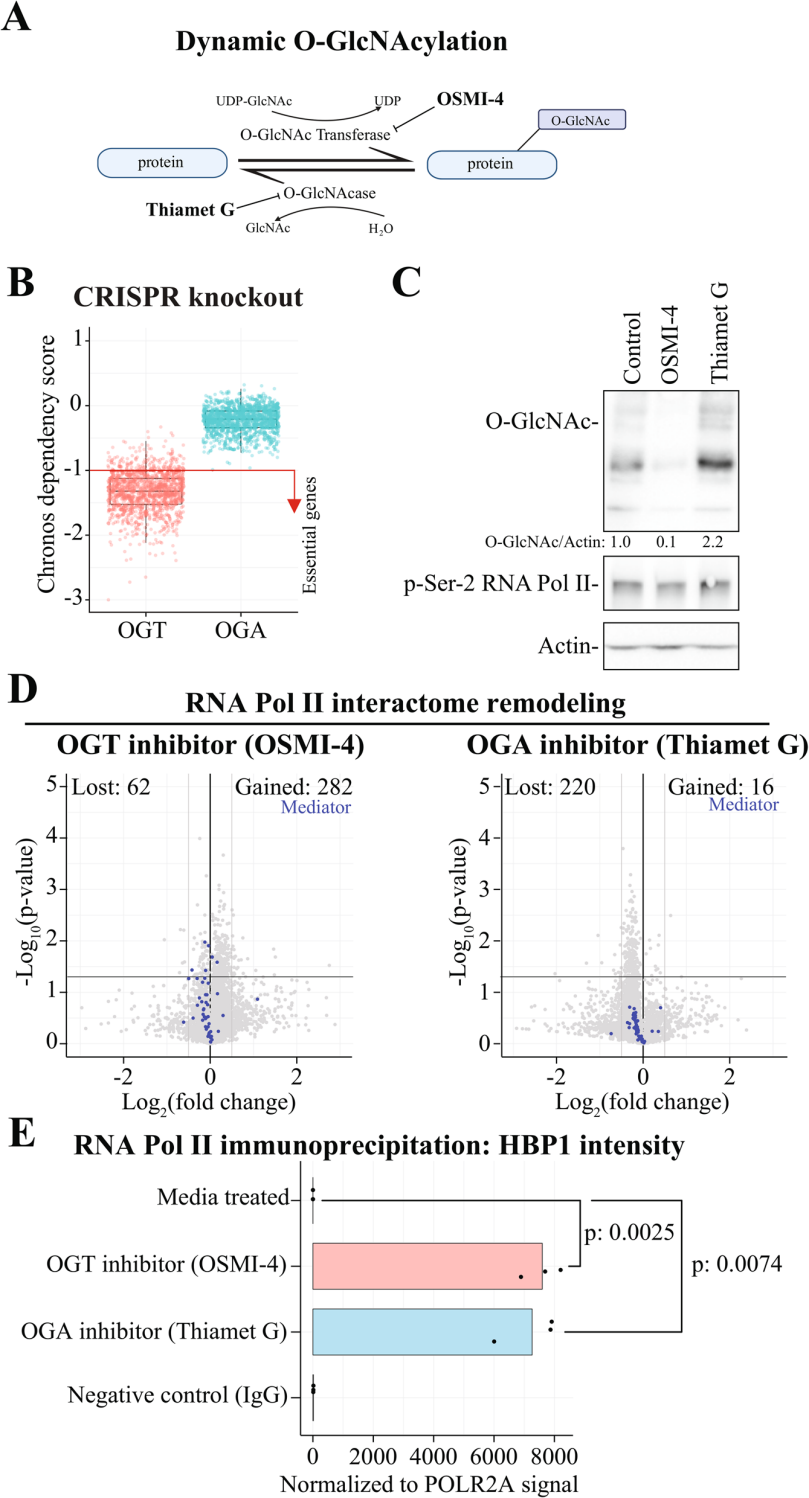
OGT inhibition stimulates protein binding to RNA Pol II. **A** O-GlcNAc transferase (OGT) catalyses the addition of O-GlcNAc group to the target proteins, and this modification is reversed by O-GlcNAcase (OGA). The activity of OGT and OGA can be inhibited with OSMI-4 and Thiamet G, respectively. **B** Data derived from the DepMap-project (https://depmap.org/portal/) shows that *OGT* is an essential gene while *OGA* is not. A score of -1 is the mean growth inhibition induced by the knockout of the common essential genes. **C** 22RV1 cells were treated with either OSMI-4 or Thiamet G (both 20 μM) for 4 h followed by protein extraction and analysis using western blot. **D** RNA Pol II-interactome is remodeled in response to OGT and OGA inhibition. 22RV1 cells were treated with OSMI-4 or Thiamet G (both 20 μM) for 4 h, RNA Pol II was immunoprecipitated and the associated proteins identified using mass-spectrometry (MS). First, the intensity values from the treated samples were normalized to the target protein (RNA Pol II). The normalized values from all the replicates are averaged to calculate the fold change relative to untreated sample. Student’s t-test was used to calculate the p-value. Proteins of a particular interest are highlighted. Please, note that the few proteins with extremely high signal are omitted from the volcano to highlight the overall effect. **E** Barplot of HBP1protein binding intensity normalized to RNA Pol II presented relative to control sample (n: 3)

O-GlcNAcylation of RNA Pol II was described in 1993 [6]; however, it is still not understood how the modification affects RNA Pol II interactome. The pair of enzymes, OGT and OGA, are responsible for all nucleocytoplasmic O-GlcNAcylation in human cells (Fig. 1A). Complete deletion of OGT is lethal (Fig. 1B), which necessitates the use of small molecule inhibitors, such as OSMI-4 [17] and Thiamet G [16], to acutely deplete and augment O-GlcNAcylation, respectively. Previously, we have shown that four hours treatment time is sufficient to deplete both overall and chromatin O-GlcNAcylation [24, 25], thus we used the same time point in this study. Indeed, we observed a fivefold decrease and an over twofold increase in the overall O-GlcNAcylation in response to inhibition of OGT and OGA, respectively (Fig. 1C). In contrast, phosphorylation of RNA Pol II was not grossly affected. These conditions reveal global changes in O-GlcNAcylation but a targeted approach is required to directly probe effects on a specific protein.

We immunoprecipitated RNA Pol II from cells treated with OGT or OGA inhibitors for four hours and identified the interacting partners using mass spectrometry (Fig. 1D). This short treatment period allows the inhibitors to act effectively while minimizing indirect effects [24–26]. First, we used western blotting to confirm that inhibition of OGT and OGA decrease and increase O-GlcNAcylation of RNA Pol II, respectively (Suppl. Figure 1A). Strikingly, inhibiting OGT activity led to a significant gain in RNA Pol II interacting proteins while inhibition of OGA activity decreased the number of interactors (Fig. 1D). These data are consistent with a model where O-GlcNAcylation and phosphorylation compete to regulate the assembly of protein complexes to the polymerase. An obvious limitation of our experimental set-up is that we do not know if RNA Pol II itself is O-GlcNAcylated or if it is the interacting proteins; however, our aim is to understand how dynamic O-GlcNAcylation affects the RNA Pol II interactome, and not whether it is the polymerase or the interacting protein that is modified.

We wanted to identify potential protein complexes that would either gain or lose interaction with RNA Pol II when either OGT or OGA is inhibited. Using systematic mapping of gained and lost interactors, we discovered that OGT inhibition increases interaction with positive regulators of transcription (RPAP1, RPAP2 and GPN1) and leads to decrease in proteins belonging to the Mediator complex (Fig. 1D, Suppl. Figure 1B and Suppl. Table 1). In contrast, only a low number of proteins bound more efficiently with RNA Pol II when OGA was inhibited, and they failed to enrich for any coherent biological processes (Suppl. Table 1). Overall, these effects, whilst being statistically significant, were of a modest magnitude.

We next explored the hypothesis that dynamic O-GlcNAcylation is required for both attraction and removal of specific proteins from RNA Pol II. To identify those proteins, we applied a stringent cut-off of either lost or gained of more that ± 2 log_2_ fold change when either OGT or OGA was inhibited. This approach identified only one protein, HMG-Box Transcription Factor 1 (HBP1), which was significantly increased by both OGT and OGA inhibitors (Fig. 1E and Suppl. Figure 1C). HBP1 regulates blood glucose and insulin concentrations through stimulating transcription of the *IGFBP1*-gene (GSE214089) but the HBP1 protein can also act as a negative regulator of transcription [27]. It is unexpected that both addition and removal of O-GlcNAcylation increases binding of HBP1 to RNA Pol II. We propose that HBP1 interacts with RNA Pol II at different stages of transcription, and addition and removal of O-GlcNAcylation is required to allow the polymerase to continue transcription. However, the lack of inhibitors to selectively target HBP1 prevented us from probing the mechanistic basis of this further.

To summarize the major findings so far, OGT inhibition resulted in a significant gain in RNA Pol II interacting proteins that positively regulate transcription. Accordingly, OGA inhibition significantly reduced the number of proteins interacting with the polymerase. These data establish the mechanistic framework of how O-GlcNAcylation regulates RNA Pol II interactome, presumably by preventing phosphorylation required to attract proteins to the polymerase. Our data pose that inhibition of OGT activity stimulates mRNA biosynthesis.

### Inhibition of either OGT or OGA stimulates mRNA maturation

We used metabolic labeling of RNA (SLAM-seq) to establish how the acute inhibition of OGT and OGA activities affect the mRNA maturation in real-time. Our proteomics approach indicated that overall mRNA synthesis is stimulated when OGT is inhibited. OGT inhibition has been shown to decrease overall levels of a high number of mRNAs [25, 28]. However, these previous studies have relied on traditional RNA-seq and relatively long treatment times, which may lead to detection of secondary effects due to pleiotropic functions OGT has [25]. To evaluate how inhibition of OGT or OGA affect the active synthesis and mRNA maturation, but exclude the potential changes in the total-mRNA pool, we had to rely on a labeling-based sequencing strategy.

**Fig. 2:**
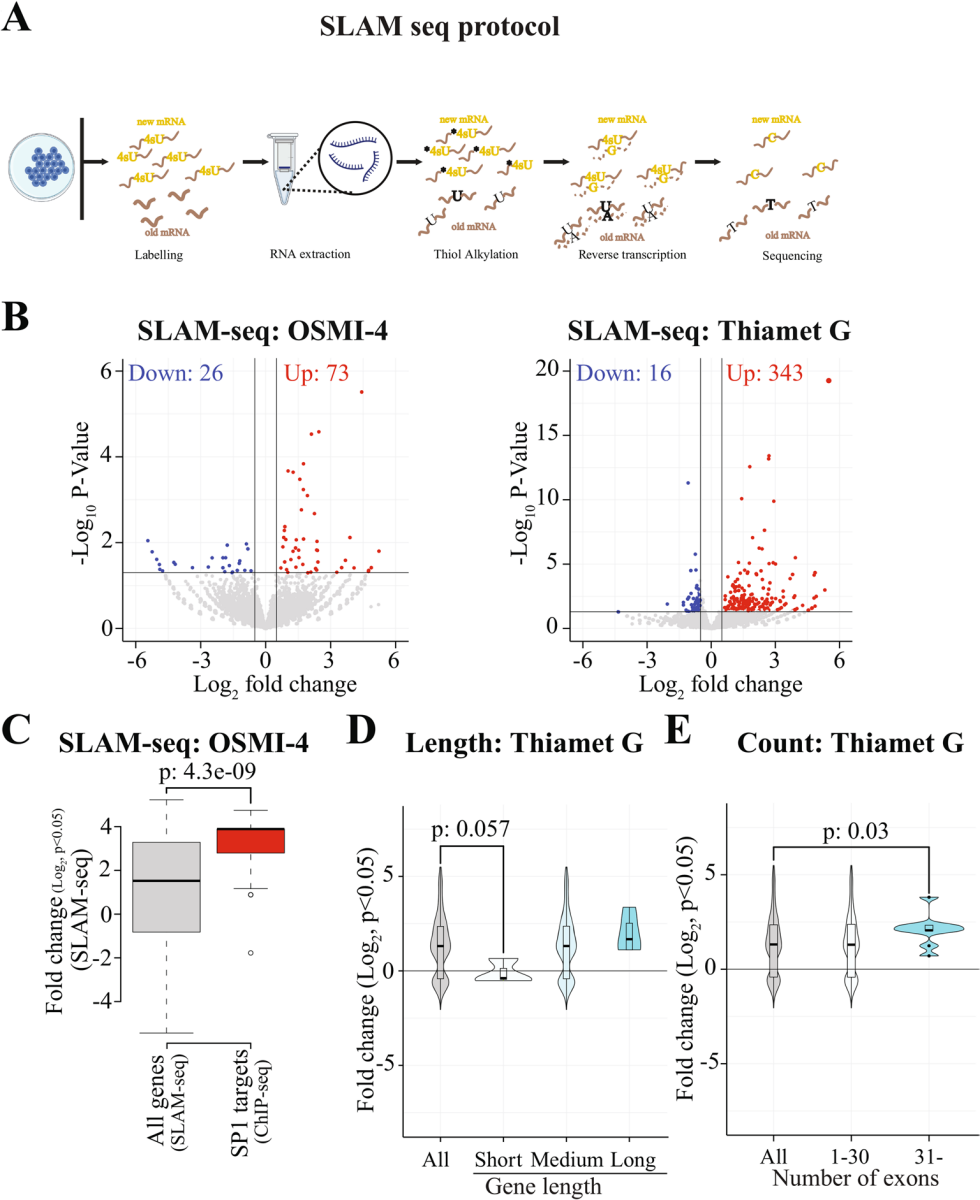
OGT and OGA are negative regulators mRNA maturation. **A** SLAM-seq protocol. Nascent transcripts are labeled with 4-thiouridine (4sU). The thiol group of the incorporated 4sU is alkylated, which is recognized as cytosine during the reverse transcription step of library preparation. Ultimately, these “cytosines” are sequenced and the number of T > C mutations are used to define the transcript as nascent in the SLAM-DUNK pipeline. **B** OGA and OGT inhibition stimulates nascent transcription. Cells were treated with OSMI-4 or Thiamet G (both 20 μM) for 4 h, samples processed using SLAM-seq protocol and analyzed using SLAM-DUNK. Significantly down- and up-regulated mRNAs are indicated in the volcano plot. **C** Box plot representing the log_2_ fold change of the SP1- and OGT-dependent genes. Publicly available ChIP-seq data (GSE266489) was used to identify the SP1 target sites. The peaks with score above 1000 were selected as the top binding sites of SP1 and the associated genes were selected for the further analysis. The red boxplot represents the SP1 target genes, which are differentially expressed in response to OGT inhibition. Student’s t-test was used to calculate the p-value. **D** OGA inhibition stimulates transcription elongation of long mRNAs. The transcriptome was trichotomized according to gene-length and the effects of Thiamet G were evaluated. Student’s t-test was used to calculate the p-value. **E** Violin plot of the number of exons and their corresponding log_2_ fold change of the differentially transcribed genes in response to OGA inhibition. Student’s t-test was used to calculate the p-value

SLAM-seq (thiol(SH)-linked alkylation for metabolic sequencing of RNA) relies on labeling of the on-going transcription with nucleotide analog, which is chemically converted rendering it recognizable as an SNP [22, 23] (Fig. 2A). After 4sU labeling and alkylation, poly(A) RNA is selected with oligo-dT beads, fragmented to ~ 200–300 nucleotides, and reverse-transcribed using oligo-dT priming to target 3′ ends [29]. This selection step means that SLAM-seq detects poly-adenylated, and therefore, mature mRNAs. Subsequently, adapters are added, and libraries are sequenced to detect T-to-C conversions, offering insights into early transcriptional dynamics. However, SLAM-seq can in principle also identify pre-maturely poly-adenylated mRNAs. To detect direct effects of an inhibitor, a short 4sU-pulse must be used. To confirm that a short labeling time results in a robust 4sU signal, we labeled cells for 10 min and compared the labeling efficiency with publicly available SLAM-seq data from the same cell line with a longer labeling time (30 min) [30]. This revealed a threefold decrease in label intensity, which is exactly the expected decrease due to the threefold shorter labeling time (Suppl. Figure 2).

Inhibition of both OGT and OGA increased the mRNA maturation as determined using SLAM-seq (Fig. 2B). To probe if these effects are reflected in the overall abundance of mRNAs, we analyzed our SLAM-seq data without the SLAM-DUNK step to generate traditional RNA-seq profiles. This approach revealed that there is minimal correlation between SLAM-seq and RNA-seq (Suppl. Figure 3). These data are in agreement with our mass spectrometry data for OGT inhibitor, where we discovered that OGT inhibition increases interaction of RNA Pol II with positive regulators of transcription (Suppl. Table 1).

However, inhibiting OGT only modestly affected mRNA maturation, and we wanted to gain more evidence for this response, and reasoned that OGT would selectively augment transcription of a specific group of genes. Earlier, we noted a modest decrease in three Mediator complex proteins from RNA Pol II (Fig. 1D, Suppl. Figure 1B and Suppl. Table 1). Out of these three proteins, MED7 and MED27 are subunits of the CRSP (cofactor required for SP1 activation) complex, and O-GlcNAcylation of SP1 inhibits its ability to stimulate transcription [31]. We established a hypothesis that OGT inhibition using OSMI-4 affects SP1-regulated transcription. To assess this, we identified the SP1 target genes from a previously reported work (GSE266489) [32] and compared how OGT inhibition affects transcription of all genes and the SP1-target genes. Indeed, OSMI-4 significantly stimulated transcription of the SP1 target genes (Fig. 2C). These data align with previous results showing that transcription decreases if the interaction between RNA Pol II and Mediator is not appropriately released [33], and identify OGT as a regulator of this step.

It was unexpected that also OGA inhibition stimulates mRNA maturation, and we wanted to better understand this effect. Earlier, a specific transcriptional kinase, CDK12, has been shown to have gene length-dependent effects on transcription [34, 35]. At a loss of how OGA regulates mRNA maturation, we asked if OGA is particularly important for the transcription of short or long genes. To probe the potential gene length-dependent effects, we trichotomized the human genes (including introns and exons) in three sets using arbitrary cut-offs in nucleotides: short (0–250), medium (251–10 000) and long (above 10 001). Interestingly, OGA inhibition stimulated transcription of genes belonging to the medium- and long-categories (Fig. 2D). In contrast, OGT inhibitor did not reveal any gene length-dependent effects (Suppl. Figure 4). To gain further evidence on the importance of high levels of O-GlcNAcylation for transcription of long genes, we asked if the transcription of mRNAs that have high exon count would be selectively increased. Indeed, OGA inhibition significantly stimulated transcription of all genes that are long or have over 30 exons (Fig. 2E and Suppl. Figure 5). These data show that high levels of O-GlcNAcylation positively regulate transcription elongation of the long and complex genes.

To summarize our major findings so far, inhibition of either OGT or OGA stimulate mRNA maturation, and these two enzymes therefore are negative regulators of transcription initiation and termination, respectively. However, the overall magnitude of the impact of either is modest, and we propose that this is because RNA Pol II can be at any position along the gene body when challenged with the inhibitors. In interpreting these results, it is important to keep in mind that our inhibitor and labeling times are relatively short, and it is therefore possible that the modest OGA inhibitor-induced decrease in transcription of the short genes might reflect an overall decrease in transcription of all genes if we inhibited OGA for a longer time. Nevertheless, OGA acts on certain proteins to negatively regulate RNA Pol II activity as manifested by the more efficient transcription of both the medium and long genes. We are yet to identify the proteins that interact more with RNA Pol II when OGA is inhibited, which presents an exciting opportunity to uncover the mechanisms underlying the stimulation of long genes. In order to characterize the position-specific interactions, it is necessary to ‘lock’ RNA Pol II at a specific stage of transcription, (initiation, promote-proximal pausing and elongation). Next, we moved on to characterize the interactome of RNA Pol II at these specific locations along the gene body.

### Biosynthesis of mRNAs in prostate cancer cells is largely independent of CDK7

**Fig. 3:**
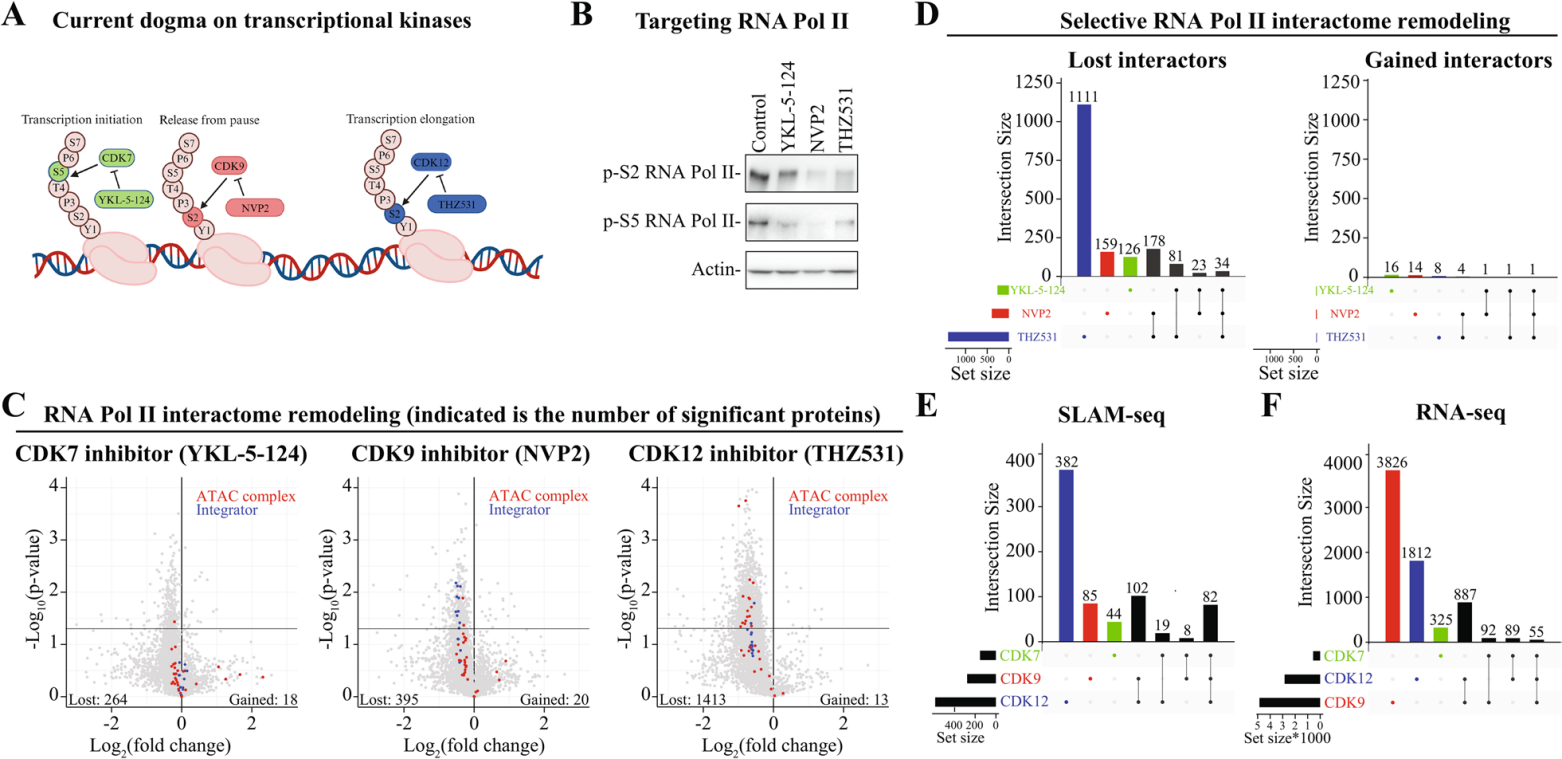
O-GlcNAcylation regulates transcription initiation and termination. **A** The current dogma positions transcriptional kinases to phosphorylate RNA Pol II at distinct positions along the gene body: CDK7 on Ser-5 to promote transcription initiation, CDK9 on Ser-2 to release RNA Pol II from promoter-proximal pausing, and CDK12 on Ser-2 to maintain the transcription on long genes. **B** Western blot showing reduced phosphorylation of RNA Pol II in response to transcriptional kinase inhibition. 22RV1 cells were treated with 500 nM YKL-5–124, 20 nM NVP2 or 500 nM THZ531 for 4 h. Data presented is representative of two biological replicates. **C** Volcano plot of RNA Pol II interacting proteins in response to inhibition of transcriptional kinases. 22RV1 cells were treated with either 500 nM YKL-5–124, 20 nM NVP2 or 500 nM THZ531 for 4 h followed by RNA Pol II immunoprecipitation. These samples were then analyzed using mass spectrometry and analyzed as in Fig. 1. ATAC- and Integrator-complex proteins are highlighted in red and blue, respectively. Significantly affected lost and gained proteins are indicated in the volcano plot. **D** Upset plot of the significantly lost and gained proteins from RNA Pol II (p-value < 0.05) in response to CDK7, 9 and 12/13 inhibition. Each of the kinases affects selective interactome of RNA Pol II. **E** Inhibition of each of the transcriptional kinases affects distinct sets of genes. 22RV1 cells were treated with 500 nM YKL-5–124, 20 nM NVP2 or 500 nM THZ531 for 4 h, labelled with 4sU for 10 min followed by RNA purification. The SLAM-seq data is analysed using SLAM-DUNK and DESeq2 pipelines to identify the differentially transcribed genes with p-value < 0.05 and log_2_ fold change (FC) ± 1. Data presented here is from two biological replicates. **F** Analysis of the SLAM-seq data with standard RNA-seq data analysis pipeline (p < 0.05 and log_2_FC ± 1)

We used specific small molecule inhibitors against each of the major transcriptional kinases, CDK7 [2], CDK9 [18] and CDK12/13 [4], to probe how inhibiting their activities affect RNA Pol II interactome and mRNA maturation using proteomics and SLAM-seq, respectively (Fig. 3A). We selected the doses from our previous paper [36] and confirmed that these doses deplete RNA Pol II phosphorylation and predominantly decrease the expression of specific target genes of each of the kinases (Fig. 3B and Suppl. Figure 6). Next, we immunoprecipitated RNA Pol II and analyzed samples using mass spectrometry. As expected, inhibition of any of the kinases reduced the number of proteins binding to RNA Pol II (Fig. 3C). We also noted that a few proteins were increasingly binding to the polymerase when the kinases were inhibited, and many of the gained interactors for CDK9 and CDK12/13 inhibitors were related to ribosome biogenesis (Suppl. Table 1). It was unexpected to see proteins involved in ribosome biogenesis to be enriched for RNA Pol II; however, RNA Pol II has been shown to ‘operate near genes encoding rRNAs to drive their expression’ [37].

To identify biological processes preferentially dependent on one of the kinases, we focused on the proteins that are selectively lost from RNA Pol II when that specific kinase is inhibited (Fig. 3D). Depletion of CDK7 activity affected a very low number of RNA Pol II interactors, and no coherent pathways that could explain the role of the kinase in regulating RNA Pol II were identified (Suppl. Table 1). Accordingly, based on SLAM-seq, only a small number of mRNAs was affected in response to CDK7 inhibition (Fig. 3E). The lack of a more robust impact was unexpected as CDK7 has been proposed to regulate transcription initiation of all genes, and thousands of genes have been reported to be affected when the activity of this kinase is perturbed [38, 39]. This conclusion is based on studies relying on traditional RNA-seq, which fails to directly measure RNA Pol II output. To establish if our cell line behaves in an unexpected manner when CDK7 is inhibited, we re-analyzed our SLAM-seq data using the traditional RNA-seq data analysis pipeline. Interestingly, in this case, we detected a four times higher number of significantly affected mRNAs when compared to SLAM-seq (Fig. 3F). To further probe this, we analyzed a CDK7 knockdown and CDK7 inhibitor treatment RNA-seq data, which again identified thousands of affected genes (Suppl. Figures 7A and 7B). These results further instill confidence on the model where inhibition of CDK7 does indeed affect the overall levels of a high number of mRNAs but likely does so without majorly affecting the mRNA maturation (Figs. 3E and 3F). We confirmed the effects of CDK7 inhibition in another prostate cancer cell line using the same CDK7 inhibitor (YKL-5–124 [2]), and also in this case, the mRNA biosynthesis of less than four hundred genes was significantly affected (Suppl. Figure 7C).

In aggregate, inhibition of CDK7 both increases and decreases the relative abundance of thousands of mRNAs with the same magnitude but the kinase is unlikely to affect RNA Pol II directly. Our SLAM-seq data were generated in prostate cancer cells after a relatively short-term inhibitor treatment, and it is possible that the effects would become more drastic over a longer time-scale or in combination with another inhibitor. Indeed, combining CDK7 and OGA inhibitors elicited effects on mRNA biosynthesis of a selective set of genes (Suppl. Figure 8). In earlier studies, CDK7 inhibition has been shown to decrease transcription using analog-sensitive derivate of the HEK cell line [33]. In the future studies, it is necessary to repeat these experiments using CDK7 inhibitors and orthogonal techniques to map the impact on the mRNA maturation. Nevertheless, the major goal in this study is to establish the crosstalk between O-GlcNAcylation and phosphorylation, and we next moved on to probe the impact of CDK9 and CDK12/13 inhibition on mRNA biosynthesis and the interactome of RNA Pol II.

### CDK9 and CDK12 activities are required to attract distinct protein complexes to RNA Pol II

**Fig. 4:**
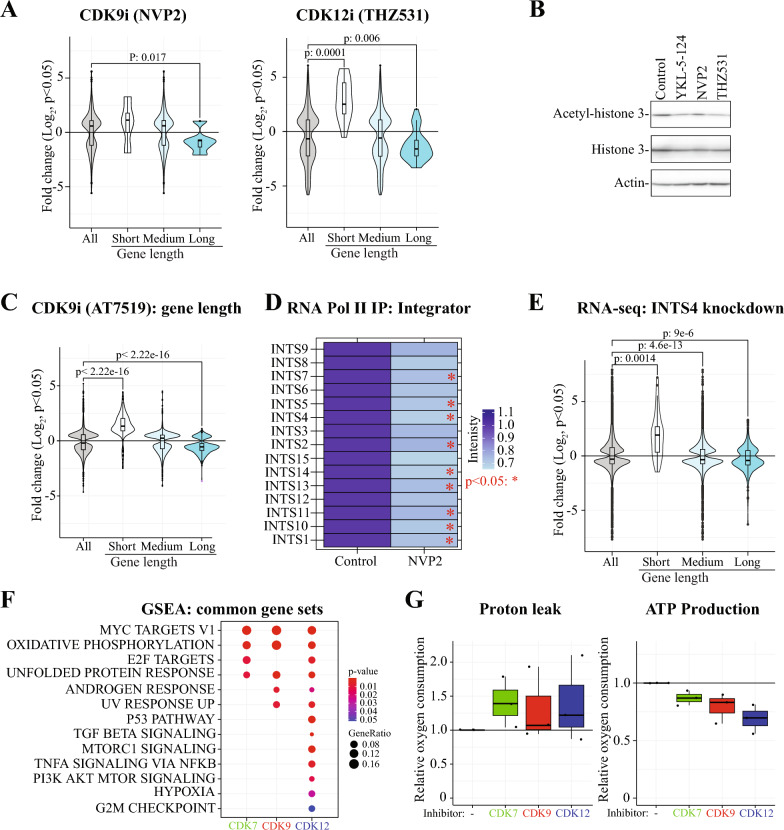
Inhibition of transcriptional kinases selectively remodels RNA Pol II interactome. **A** Inhibition of either CDK9 or CDK12/13 stimulates transcription of the short genes and suppresses transcription of the long genes. The differentially labelled transcripts in response to CDK9 and CDK12/13 inhibition were trichotomized according to the gene-length. Student’s t-test was used to calculate the p-value. **B** Western blot showing reduced acetylation of histone 3 predominantly in response to CDK12/13 inhibition. 22RV1 cells were treated with 500 nM YKL-5–124, 20 nM NVP2 or 500 nM THZ531 for 4 h followed by protein extraction. Data presented is representative of two biological replicates. **C** LNCaP cells were treated with CDK9 inhibitor (0.5 μM AT7519) for four hours followed by RNA extraction and RNA-seq. This is re-analysis of data reported earlier (GSE116778). The differentially expressed genes in response to CDK9 inhibition were trichotomized according to the gene-length. Student’s t-test was used to calculate the p-value. **D** Integrator complex proteins are selectively lost from RNA Pol II when CDK9 is inhibited. Heatmap of integrator complex protein intensity values first normalized to RNA Pol II-signal and then presented relative to control. **E** The loss of the integrator complex protein INST4 stimulates the transcription of short genes, mimicking the effects of the CDK9 inhibition. To evaluate the effects, we used the gene-expression table deposited on GEO to call the DEGs (p < 0.05 and log_2_FC ± 1) from the INST4 knockdown RNA-seq data (GSE125534) and evaluated the impact on DEGs according to gene-length. Students t-test was used to calculate the p-value. **F** Gene set enrichment analysis of the differentially transcribing genes in response to CDK7, CDK9 or CDK12/13 inhibition.** G** Mitochondrial oxygen consumption rate (OCR) was measured using the Seahorse instrument. Prior to the Seahorse-assay, cells were treated with inhibitors of the transcriptional kinases for 4 h. Data is from three biological replicates each having four technical replicates

Our SLAM-seq data revealed that inhibition of either CDK9 or CDK12/13 stimulates maturation of short mRNAs and suppresses maturation of the particularly long mRNAs (Fig. 4A). CDK12/13 inhibition has been previously shown to cause these effects due to release of the negative regulator of CDK9 and intronic polyadenylation, respectively [34, 40, 41]. We confirmed that CDK12/13 inhibition decreases interaction of the negative regulators of transcription with RNA Pol II (‘Mixed, incl. 7SK snRNP binding, and nucleoplasmin family’, Supp. Table 1) and validated the increase in intronic poly-adenylation also in prostate cancer cells (Suppl. Figure 9). Further exploration of the protein complexes selectively depleted from RNA Pol II identified protein machineries involved in histone acetylation, such as the ATAC complex (Fig. 3C and Suppl. Table 1). This should have severe consequences on the cells’ ability to maintain histone 3 acetylation on the transcriptionally active chromatin. Indeed, CDK12/13 inhibition decreased histone 3 acetylation (Fig. 4B). We hypothesized that decrease in CDK12/13 activity would render cells addicted on the genes forming the ATAC complex in the situation where RNA Pol II interaction with the complex is reduced. To test this hypothesis with existing clinical data, we identified prostate cancer patient samples with inactivation of the *CDK12*-gene from the TCGA, Firehose Legacy [42] and SU2Cancer datasets [43]. This approach indeed revealed mutual exclusivity between *CDK12*-inactivation and deletions in the genes encoding for the ATAC complex members (Suppl. Figure 10).

It was striking that inhibition of CDK9 stimulates transcription of short genes, and we wanted to better understand this response. To do this, we used previously published RNA-seq data [25] and confirmed that CDK9 inhibition indeed stimulates transcription of the short genes and decreases transcription of the long genes also in another model system (Fig. 4C). These data add to the functionality of CDK9, as the kinase has been previously shown to regulate RNA Pol II promoter-proximal pause-release [44–46]. Gressel *& al.* (2017) revealed that inhibiting CDK9 leads to increase of RNA Pol II on the transcription start sites and decrease later on along the gene body [46]. This can be interpreted as an increased pausing as proposed by the authors, or, by increased initiation of transcription of the short genes, if the data was analyzed according to the gene length. Nevertheless, we show in two different model systems using both SLAM-seq and RNA-seq that CDK9 inhibition stimulates transcription of short genes, and we reasoned that the protein machineries selectively depleted from RNA Pol II can explain this effect.

The interaction of the Integrator complex proteins with RNA Pol II was significantly decreased in response to CDK9 inhibition (Figs. 3C, 4D and Suppl. Table 1). We note that the overall magnitude of the effect was relatively modest, ranging from 15 to 35% reduction; however, this effect was consistent across 15 proteins belonging to Integrator. The Integrator is involved in multiple aspects of RNA Pol II biology, and it can both positively and negatively regulate transcription elongation [47–49]. Earlier, it has been established that Integrator interacts with the negative elongation factor (NELF) [50], and CDK9-dependent phosphorylation facilitates dissociation of NELF from RNA Pol II in vitro to support formation of the transcription elongation complex [51]. Stadelmayer *& al.* reported that integrator subunit INTS11 is required for the transcription elongation of genes that exhibit high levels of NELF binding to the promoters, which implies that a specific subset of the genes is dependent on the Integrator complex for their transcription [50]. To probe if Integrator is a gene length-dependent regulator of a specific set of genes, we analyzed previously generated data where the authors depleted INTS4 or INTS11 using a short hairpin RNA-based strategy and performed RNA-seq. Indeed, depletion of INTS4 significantly stimulated transcription of the short genes and similar effect was also observed for INTS11 (Figs. 4E and Suppl. Figure 11A). On the other hand, we noted only a modest effect on the long genes when either was depleted. In the case of CDK12/13 inhibition, the increase in intronic poly-adenylation explains the decrease in transcription of the long genes (Suppl. Figure 9A). However, CDK9 inhibition did not increase intronic poly-adenylation (Suppl. Figure 11B). We therefore propose that defective transcription maturation of the long mRNAs in response to CDK9 inhibition is due to multiple effects that include defects in the release from promoter-proximal pausing, determination of the appropriate poly-adenylation site, and potentially also other processes.

To summarize our major findings so far, we identified selective functions for CDK9 and CDK12/13, which regulate assembly of the Integrator to RNA Pol II and maintain histone 3 acetylation, respectively. We note that a large number of proteins were commonly lost when either CDK9 or CDK12/13 were inhibited, proposing that these kinases regulate somewhat overlapping aspects of RNA Pol II interactome (Fig. 3D). When interpreting these results, it is important to keep in mind that inhibition of OGT or OGA may alter the activity of kinases, while inhibition of any of the kinases may cause O-GlcNAcylation or its removal; therefore, our results reveal how inhibition of each of the factors alter RNA Pol II interactome and activity, but these effects may also depend on other post-translational modifications. Previously, it has been established that inhibition of OGT affects global phosphorylation [26], and we note that inhibition of particularly CDK9 and CDK12/13 increase global O-GlcNAcylation (Suppl. Figure 12).

We moved on to identify the biological processes that are activated in response to inhibition of each of the major transcription kinases. Unexpectedly, despite affecting largely varying number of distinct sets of mRNAs, inhibition of any of the major transcriptional kinases, CDK7, CDK9 or CDK12/13, activated the same biological response, oxidative phosphorylation, based on gene set enrichment analysis (Fig. 4F). We have previously shown that CDK9 inhibition decreases ATP production after a longer treatment time, but this effect could also be due to effects on cell viability [52, 53]. To directly assess if inhibition of CDK7, CDK9 or CDK12/13 affect mitochondrial activity after four hours of treatment, we relied on the Seahorse assay, which measures the mitochondrial oxygen consumption rate (OCR) of the mitochondrial electron transport chain. Interestingly, inhibition of any of the major transcriptional kinases increased proton leak and suppressed mitochondrial ATP production (Fig. 4G). Currently, it remains unclear if the effects on mitochondrial activity are due to transcriptional effects or by directly modulating mitochondrial activity through a specific substrate. However, we turned our focus back to the major point of our study, the regulation of RNA Pol II activity by O-GlcNAcylation and phosphorylation.

### OGA is required for mRNA maturation when transcription elongation is defective

**Fig. 5:**
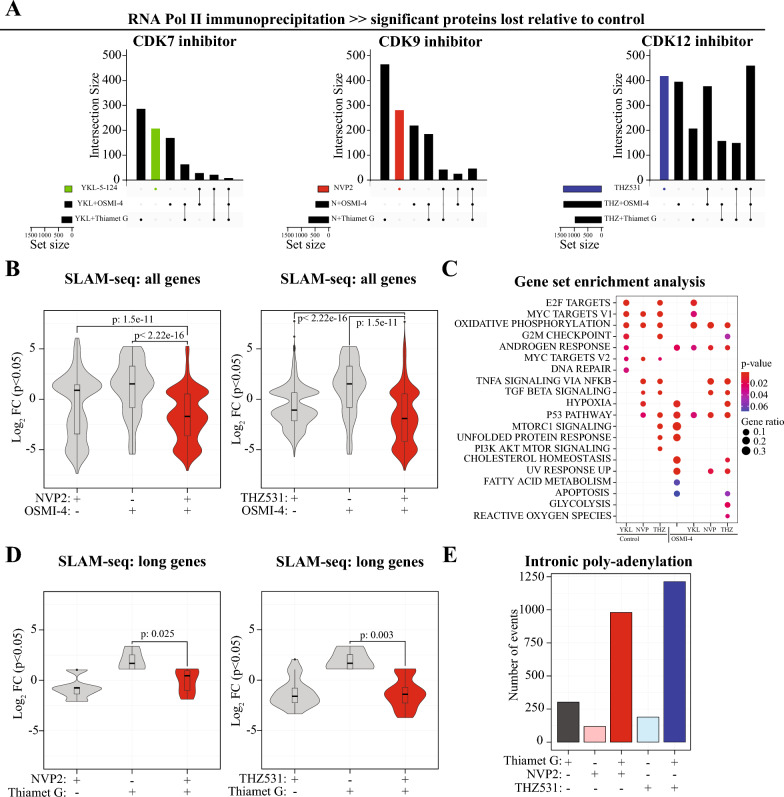
Inhibition of OGA leads to defects in RNA maturation due to intronic polyadenylation. **A** Loss of CDK12/13 activity drastically affects the RNA Pol II interactome. 22RV1 cells were simultaneously treated with the transcriptional kinase inhibitor with and without 20 µM OSMI-4/Thiamet G for 4 h, followed by RNA Pol II immunoprecipitation. These samples were analyzed using mass spectrometry. The raw intensity values were first normalized to RNA Pol II and the fold change was calculated relative to untreated sample. Student’s t-test was used to calculate the significance from three biological replicates. **B** Combined inhibition of CDK9 or CDK12/13 and OGT leads to further suppression of mRNA biosynthesis. 22RV1 cells were treated with 20 nM NVP2 or 500 nM THZ531 and 20 µM OSMI-4 for 4 h, labelled with 4sU for 10 min followed by RNA purification. The SLAM-seq data was analysed using SLAM-DUNK and DESeq2 pipelines to identify the differentially transcribed genes with p-value < 0.05 and log_2_FC ± 1. Data presented is from two biological replicates. Student’s t-test was used to calculate the p-value. **C** Gene set enrichment analysis of the DEGs identified from the SLAM-seq data showing the loss of MYC-driven transcription when OGT inhibitor is combined with both the CDK9 and CDK12/13 inhibitors. **D** O-GlcNAcylation rescues the long genes from the effects of the CDK9 inhibitor (n = 2). **E** Barplot depicting the total number intronic poly-adenylation sites in the indicated conditions

We generated mRNA maturation profiles (SLAM-seq) and RNA Pol II interactome maps of cells simultaneously treated with compounds targeting CDK7, CDK9 or CDK12/13 along with OGT and OGA inhibitors. Our RNA Pol II proteomics data revealed that CDK12/13-dependent phosphorylation is required for the recruitment of a much higher number of proteins than CDK7- or CDK9-dependent phosphorylation, both in conjunction with dynamic O-GlcNAcylation, and in the absence of it (Fig. 5A). The RNA Pol II proteomics data imply that transcription should be severely compromised when inhibitors of transcriptional kinases and either OGT or OGA are combined.

Perturbation of OGT and CDK9 or CDK12/13-dependent phosphorylation resulted in a drastic decrease in the mRNA maturation when compared to targeting OGT and CDK7 as determined using SLAM-seq (Fig. 5B and Suppl. Figure 13). Combinations of OGT inhibitor with compounds targeting CDK7, CDK9 or CDK12/13 did not have gene length-dependent effects but they all affected the largest group of genes ‘medium’ (Suppl. Figure 13). Next, we asked if OGT is required by cells to trigger an adaptive transcriptional response using the gene set enrichment analysis. This approach revealed that OGT inhibition prevents activation of MYC-signaling induced in response to targeting of either CDK9 or CDK12/13 (Fig. 5C). These effects may in part be explained by the negative regulator of MYC signaling, HBP1 [54], which is recruited to RNA Pol II when OGT is inhibited (Fig. 1E). Given the similar transcriptional effects for both kinases, we examined the proteins commonly lost when CDK9 or CDK12/13 are targeted in combination with OGT inhibition. These proteins were involved in transcription elongation from RNA Pol II promoters (Suppl. Table 1). In contrast, the protein machineries that we earlier found to be dependent on CDK9- or CDK12/13-dependent phosphorylation, the Integrator and the ATAC complexes, respectively, were still significantly lost from RNA Pol II when OGT inhibitor was added together with the perspective kinase inhibitors (Suppl. Figure 14). These data show that a subset of protein machineries depend on phosphorylation while others are regulated by both phosphorylation and O-GlcNAcylation. Our SLAM-seq and RNA Pol II proteomics data explain the previous finding that OGT inhibition sensitizes cells to compounds targeting CDK9 [25] and CDK12/13 [55], which we also confirmed here (Suppl. Figure 15). Our data show that OGT is required to both sustain overall transcription and to mount an adaptive response to both CDK9 and CDK12/13 inhibition.

We reasoned that OGA inhibition can rescue transcription and transcriptional program from compounds targeting CDK9 or CDK12/13 because OGA inhibition augments O-GlcNAcylation. Accordingly, gene set enrichment analysis revealed that depleting OGA activity did not affect cells ability to induce MYC signaling in response to CDK9 or CDK12/13 inhibition (Suppl. Figure 16). In these cases, HBP1 did not interact with RNA Pol II significantly more (data not shown). Earlier, we demonstrated that inhibition of either CDK9 or CDK12/13 leads to a robust suppression of long genes (Fig. 4A) and we therefore asked if OGA inhibition rescues transcription of long genes. Indeed, OGA inhibitor rescued transcription of the long genes in response to CDK9 inhibition and also modestly for the CDK12/13 inhibition (Fig. 5D). In addition, OGA inhibition rescued transcription of the medium length genes when CDK12/13 was inhibited (Suppl. Figure 17). We failed to explain these results through our proteomics data, and therefore reasoned that the lack of rescue of the long genes is due to premature poly-adenylation when inhibitors of OGA and CDK12/13 are combined, which we confirmed to be the case (Fig. 5E). When either OGT or OGA was inhibited together with CDK12/13, assembly of proteins involved in pre-mRNA binding to RNA Pol II was defective (Suppl. Table 1). Accordingly, despite seemingly rescuing the transcription of genes after targeting CDK9 and CDK12/13, OGA inhibition sensitized cells to compounds targeting CDK9 or CDK12/13, which can atleast in part be explained due to premature poly-adenylation (Suppl. Figure 18).

In this study, we show that OGT and OGA negatively regulate transcription initiation and elongation, respectively, and our work proposes dynamic O-GlcNAcylation as a quality control mechanism operating in conjunction with RNA Pol II phosphorylation. Our data also proposes that CDK9 functions as a *bona fide* transcription elongation kinase but serves different functions than the previously established transcription elongation kinases CDK12/13. Finally, our results and the existing literature fail to describe the kinase responsible for transcription initiation, and due to the critical nature of this step, we propose that it can be catalyzed by redundant factors that may differ between cell types.

## Discussion

To summarize the major findings from the comprehensive mapping of the RNA Pol II interactome and mRNA maturation profiling, our data identify OGT and OGA as negative regulators of mRNA biosynthesis. Furthermore, our results propose that high activity of the transcription elongation kinase CDK12/13 is critical to maintain transcriptionally active chromatin-status. Our data underscore the need to directly measure RNA Pol II activity rather than relying on the overall mRNA abundance to identify direct functional effects.

Composition of the transcription machinery was initially established through protein purification-based approaches, which led to identification of kinase-activities that can phosphorylate RNA Pol II. These assays determined that CDK7 can phosphorylate RNA Pol II CTD on ser-5 but it fails to phosphorylate RNA Pol II that is already engaged with the promoter sequence [56]. CDK9 was initially identified as PITALRE due to its homology with cell division cycle 2 (CDC2) family of kinases, and later on it was shown to phosphorylate RNA Pol II on Ser-2 [57–59]. In the biochemical studies where the activities of CDK9 and CDK12 have been described, both of them phosphorylate Ser-2 and Ser-5 [59, 60]. Combined with existing literature and our SLAM-seq results describing the lack of impact of CDK7 inhibition, it is possible that OGT, CDK9 and / or CDK12 can replace CDK7 to promote Ser-5 phosphorylation, particularly when the activity of CDK7 is depleted.

In this study, our strategy was to systematically map RNA Pol II interactome after short time depletion of the activities of the major transcriptional kinases using selective inhibitors. We show that high activity of CDK9 is pivotal for the efficient transcription elongation of the particularly long genes, while inhibition of its activity stimulates transcription of the short genes by regulating the assembly of Integrator complex to RNA Pol II (Figs. 4A, C, D). Our data contrast the notion that CDK9 only releases RNA Pol II from the promoter-proximal pausing, as has been proposed in the literature [61, 62], but rather propose that low CDK9 activity favors transcription of genes that are short due to remodeling of RNA Pol II interactome. It is possible that CDK9 serves a distinct function in these very short genes. The unexpected implication of our data using CDK inhibitors is that a very modest amount of RNA Pol II interacting proteins is sufficient for generation of polyadenylated mRNAs: inhibition of CDK7, CDK9 or CDK12/13 robustly deplete the vast majority of proteins from RNA Pol II, yet cells are able to support transcription based on SLAM-seq (Figs. 3C, E).

The fifth amino acid in the CTD, Ser-5, emerges as the major site that is targeted by the transcriptional kinases and OGT [6, 7, 59, 60]. Earlier, using partially purified components, it has been established that O-GlcNAcylation is required during transcription initiation [7, 8] and the modification is also associated with the proteins regulating transcription elongation [15]. Our data identify OGT as a negative regulator of mRNA biosynthesis, and we propose that the enzyme acts at multiple steps during transcription (initiation, elongation and termination), but has relatively modest effect at any.

RNA Pol II activity is regulated particularly during the pre-initiation complex formation and promoter clearance, and both of these steps involve an elegant interplay between positive and negative regulators. However, establishing the role of a post-translational modification on RNA Pol II activity is not a trivial task because even the minimal RNA Pol II transcription system consists of the polymerase and several general transcription factors (including TFIIB, -D, -E, -F, and -H), each having typically multiple polypeptides [63–65]. Many of these proteins are substrates for OGT, OGA and transcriptional kinases, which makes it difficult to draw conclusions on what particular modification has the causal effect on the observed outcome. However, certain conclusions can be drawn from our work here and the literature, even though many reports infer effects on RNA Pol II by measuring proteins on the chromatin and whole cell phospho-proteomics, which should not be directly attributed to effects of a kinase on polymerase. Existing data indicate that CDK7 promotes release of RNA Pol II from Mediator (indirect evidence) [33], CDK9 promotes dissociation of negative elongation factors from RNA Pol II [61], while CDK12/13 are important to recruit the splicing machinery (based on indirect evidence using phospho-proteomics) [66].

Existing literature relying on in vitro assays propose that RNA Pol II O-GlcNAcylation is required for the promoter entry of the polymerase [8], occurs during pre-initiation complex formation and must be removed in an ATP-dependent manner before the polymerase continues to elongation [7, 8]. In in vitro pausing assays, addition of recombinant OGT results in pausing [67]. Interpretation of the data from these assays has the same issue as other experiments involving more than one protein: it is not possible to know what the targets of OGT and OGA are to cause the observed effect. In addition, these assays rely on partially purified components, and addition of a recombinant protein may lead to a skewed relative abundance between different components. It is peculiar that O-GlcNAcylation emerged relatively recently in evolution as a regulator of RNA Pol II, and the polymerase is capable of entering the promoters in the absence of O-GlcNAcylation for example in yeast, which does not have OGT at all. Nevertheless, both our data and existing literature establish that OGT and OGA act at multiple points during mRNA biosynthesis.

We propose that, in contrast to phosphorylation mediated by CDK7, CDK9 and CDK12/13 that predominantly positively regulate transcription, the impact of OGT and OGA is distinct depending on what proteins are targeted and at what stage along the gene body. For example, O-GlcNAcylation is important for promoter loading of RNA Pol II, but the modification must be removed before the polymerase can leave the promoter [7, 8]. However, OGA activity is required for efficient transcription elongation based on both our results here and previous literature [15]. The role of OGT and OGA later on in transcription may represent an alternative mechanism to support transcription; according to this proposal, we have earlier demonstrated that inhibition of CDK9 induces hyper-O-GlcNAcylation of CDK9 itself and also increases the overall chromatin O-GlcNAcylation [15, 36].

In the future, it becomes critical to identify how and where the different glyco-forms of RNA Pol II operate. These experiments necessitate the development of tools that currently do not exist, in particular, antibodies that recognize the specifically O-GlcNAcylated aminoacid on the RNA Pol II CTD. In this work, we have predominantly focused on the basic molecular mechanisms regulating RNA Pol II, but it has not escaped our notion that these discoveries open direct avenues to actionable synthetic (inactivation of the *CDK12*-gene in specific cancers) and combinatorial lethalities.

## Conclusions

We identify both addition and removal of O-GlcNAcylation as negative regulators of mRNA biosynthesis and propose that the dynamic O-GlcNAcylation serves as quality control to assure successful generation of mature mRNAs. Our work identifies unprecedented redundancy in the regulation of RNA Pol II, which increases resilience towards transcriptional stress. Based on these results and literature, we propose that combinatorial targeting, or cancer cell-specific defects, establish an actionable avenue to selectively kill the malignant cells using compounds interfering with transcription.

## Materials and methods

### Experimental model details

LNCaP, 22RV1 and C4-2 cell lines were obtained from the American Tissue Culture Collection. Cells were maintained in RPMI medium supplemented with 10% fetal bovine serum (FBS). For androgen-starvation experiments, cells were kept in phenol red-free RPMI supplemented with charcoal-stripped FBS for three days prior to treatments.

### Preparation of cell lysate for western blot

Cell lysates for western blotting were prepared as previously described [68]. All the steps are performed at 4 °C, unless otherwise mentioned. In brief, the cells were collected in PBS by centrifuging at 3 000 rpm for 5 min. The RIPA lysis buffer was supplemented with proteinase, phosphatase and O-GlcNAcase (Thiamet G) inhibitors. The cells were lysed for 30 min, centrifuged at 14 000 rpm for 5 min and the supernatant was collected. Protein concentration was determined with bicinchoninic acid (BCA) assay.

### Sample preparation for RT-PCR

RNA was isolated using Amersham RNAspin Mini Kit (Cytiva) according to the manufacturer’s instructions. The cDNA used for qPCR was prepared using qScript cDNA Synthesis Kit (Quanta Biosciences). Primer sequences are provided in the supplementary Table 2.

### Immunoprecipitation

22RV1 cells were allowed to adhere to the plates for a day and treated with the inhibitors of either CDK7, CDK9, CDK12 alone or in combination with OGT/OGA inhibitors for four hours. Unless otherwise mentioned, cells were kept on ice and buffers used were ice cold. Next, the cells were washed with PBS and collected by centrifugation at 1 000 rpm for 10 min. The cells were solubilized into 600 µl of lysis buffer (50 mM Tris–HCl, pH 7.4, 2 mM EDTA, and 300 mM NaCl), supplemented with the protease, the phosphatase and the OGA (Thiamet G) inhibitors and incubated for 30 min. The cells were then centrifuged at 15 000 rpm for 10 min, the supernatant was collected, and the centrifugation was repeated for the supernatant. Next, 1 200 µl of dilution buffer (50 mM Tris–HCl, pH 7.4 and 2 mM EDTA) was added to the samples to achieve final conditions of 100 mM NaCl, 50 mM Tris–HCl, pH 7.4, 2 mM EDTA. Next, the cell lysates were incubated overnight on the rotator at 4 °C with anti-RNA Pol II antibodies (2 µg of each sc-56767 and sc-47701). Next day, 60 µl of protein A/G magnetic beads were washed three times with washing buffer, beads were added to the cell lysate / antibody solution, which was already incubated overnight, and incubation was continued for 2 h. After this, the samples were washed three times with washing buffer (50 mM Tris–HCl, pH 7.4, 2 mM EDTA, and 100 mM NaCl), once with PBS, 20 µl of PBS was added to each sample, and the samples were frozen in dry ice. Samples were stored in -80 °C until mass spectrometry-analysis (purchased as service from University of Turku).

### Mass spectrometry data analysis

We modified the analysis method from Harlen *& al.* [69]. First, we identified the proteins having no intensity values and pseudo coded those to the value of 1. Second, for individual replicates, the signal intensity of the identified proteins were normalized to the signal intensity of the immunoprecipitation target protein (RNA Pol II) to account for the potential differences in the immunoprecipitation efficiency. Third, average intensity values were calculated from three biological replicates, which were used to calculate the fold change relative to the control sample. Student’s t-test was used to evaluate the significance. The proteins having p-value < 0.05 and fold change more than or less than zero were considered as gained or lost interactors, respectively.

### SLAM-seq

22RV1 cells were allowed to adhere to the plate for one day. Cells were treated for four hours with the inhibitors of CDK7 (YKL-5–124), CDK9 (NVP2) or CDK12 (THZ531) in combination with either OGT (OSMI-4) or OGA (Thiamet G) inhibitor. Followed by 4-thiouridine (1 mM) pulse for ten minutes. Subsequently, RNA was collected using Amersham RNAspin Mini Kit (Cytiva) as per manufacturer’s instructions, except 0.1 mM dithiotreitol (DTT) was added to all the buffers in the RNA extraction kit to protect the thiol group of the added nucleotide analogue 4-thiouridine. Alkylation was performed as previously described by Herzog *& al* [70]*.* and Lexogen manual, with some modifications. In brief, the alkylation reaction was performed for 15 min at 50 °C and the reaction mixture consisted of 500 nG RNA, 10 mM iodoacetamide, 50 mM NaPO_4_ (pH 8.0). Alkylation reaction was terminated by adding 1 µl of 1 M DTT. After this, RNA was purified again using ethanol precipitation. In brief, 5 µl NaOAc (3 M pH 5.2) and 125 µl 100% ethanol were added to solution, samples vortexed and placed to -80 °C for 30 min. Next, samples were centrifuged for 30 min at 14 000 rpm at + 4 °C, washed with 75% ethanol, and centrifuged again for 10 min. Most of the ethanol was removed, the remaining allowed to evaporate for 10 min, after which RNA was solubilized into nuclease-free water. SLAM-seq libraries were prepared using QuantSeq 3’ mRNA-Seq library preparation kit (FWD) for Illumina sequencing and sequencing was performed using SR100 High Output sequencing on Illumina NextSeq 2000. Library preparation and sequencing were bought as a service from FIMM Genomic Services, Finland.

### SLAM-seq data analysis

Single end fastq files were subjected to quality control using FastQC. The SLAM-seq data is analysed using SLAM-DUNK pipeline [23], and was run here as default. The count tab-delimited file was used to generate the count matrix. All the TC-reads were RPMu normalized before calling the differentially labelled transcripts (DLTs) using DESeq2 [71]. Briefly, for normalization, TCreadCount of each transcript was divided by the sum of the non-TC reads and multiplied by 10 000 000. The significant DLTs were filtered with the threshold of p-value < 0.05 and log_2_FoldChange ± 1. Gene set enrichment analysis was performed using clusterProfiler [72] in R. For the gene length analysis, gencode.v29.annotation.gtf was used to identify the gene length of each transcript. The transcripts having length ranging from 0 to 249 bp, 250–10 000 bp and above 10 000 were categorized as short, medium, and long, respectively.

To analyze the SLAM-seq data for the overall gene-expression profile (as done in traditional RNA-seq), the fastq files were aligned using Bowtie2 with default parameters and corresponding index files were generated. These files were used for calling the differentially expressed genes (DEGs) using DESeq2 [71]. Same thresholds were used to filter the significant DEGs as used for SLAM-DUNK. Volcano plots were generated using ggplot2 in Rstudio v 2023.03.0 + 386.

### Intronic polyadenylation site identification

We used IPAfinder to identify the intronic polyadenylation sites (IPA) from the RNA-seq data [73]. In this study, we have used the population-level IPA detection and quantification, as we were interested in the counts of the IPA per samples and not the differential IPA usage. In brief, all the SLAM-seq raw fastq files were aligned with STAR aligner as it is a splice site aware algorithm. Using the “IPAFinder_Population.py”, all the IPA events from the samples were identified. The output of IPAFinder is a tab-delimited text file in which each line has an identified IPA event and their corresponding features. In the figures, we have plotted the IPA-score assigned to the predicted IPA-event or the total number of IPA events.

### ChIP-seq data analysis

Raw fastq files were downloaded from the Gene Expression Omnibus database. The files were first subjected to quality control using FastQC. Fastq files were aligned using Bowtie2 with default parameters. Duplicate reads from the aligned BAM files were removed using MACS2 [74] filterdup module. The filtered BAM files were subjected to peak calling using MACS2 callpeak module with default parameters. BigWig files and the metagene plots were generated using deeptools [75].

### Seahorse mito stress test

Metabolic flux analysis was performed using the Agilent Seahorse Xfe96 Analyzer. The cells were seeded onto Seahorse Xfe96 microplates in RPMI + 10% FBS two days before the experiment. Prior to the assay, the growth medium was replaced with Seahorse assay medium. This medium is free of bicarbonate, has low buffer capacity, and contains no phenol red. Before use, we added 1 mM pyruvate, 2 mM glutamine, and 10 mM glucose to the assay medium. Next, the cells were incubated in the CO_2_-free incubator for 60 min. During the Seahorse-assay, inhibitors of the mitochondrial respiratory chain are injected at specific time points to assess different aspects of the mitochondrial function. Oligomycin inhibits ATP synthase, allowing the measurement of ATP-linked respiration. FCCP uncouples mitochondrial respiration from ATP production, providing a maximal respiration rate. Rotenone/antimycin A block the electron transport chain, allowing the measurement of non-mitochondrial respiration. The Seahorse Wave cloud version was used to analyse the data and the plots were made using Rstudio.

## Supplementary Information

Supplementary material 1.

Supplementary material 2.

Supplementary material 3.

## Data Availability

This study did not generate new unique reagents or original code. Any additional information / custom scripts required to re-analyze the data reported in this paper are available from the lead contacts upon a reasonable request. All the SLAM-seq data has been deposited to SRA with the following accession numbers: PRJNA1108610 and PRJNA1118101.
