# Supplementary material for: Dynamic O-GlcNAcylation and phosphorylation attract and expel proteins from RNA polymerase II to regulate mRNA maturation
Source: J Biomed Sci. 2025 Apr 4;32:39. doi: 10.1186/s12929-025-01135-9 (PMC11969731; doi:10.1186/s12929-025-01135-9)
Supplement: Supplementary file 1 — Supplementary material 1. [file 12929_2025_1135_MOESM1_ESM.pdf]

Supplementary figures, supplementary figure legends and supplementary table legends.

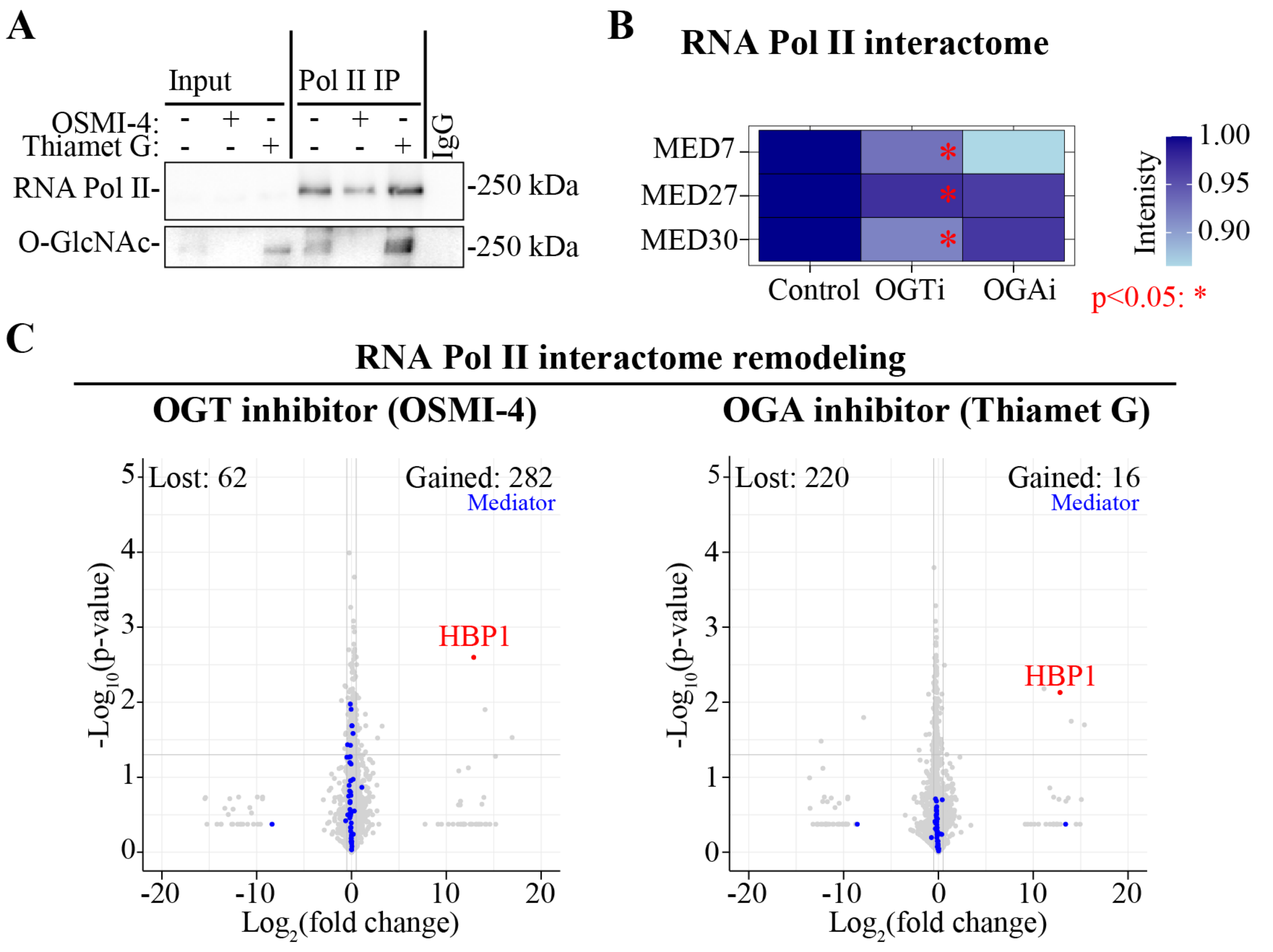

Supplementary figure 1. Inhibition of OGT and OGA affects RNA Pol II complex composition. A) OGT inhibitor decreases RNA Pol II O-GlcNAcylation while OGA inhibition increases RNA Pol II O-GlcNAcylation. 22RV1 cells were treated for 4 hours with fresh media, 20 µM OGT inhibitor OSMI-4, or 20 µM OGA inhibitor Thiamet G, and immunoprecipitation was performed. **B)** Mediator complex proteins lose their interaction with RNA Pol II in response to OGT inhibition. Heatmap depicting RNA Pol II normalized intensity values of the mediator complex proteins relative to control from the OGT and OGA inhibitor treated samples. **C)** RNA Pol II-interactome is remodeled in response to OGT and OGA inhibition. First, the intensity values from the treated samples were normalized to the target protein (RNA Pol II). The normalized values from all the replicates are averaged to calculate the fold change relative to untreated sample. Students t-test was used to calculate the p-value. Proteins of a particular interest are highlighted.

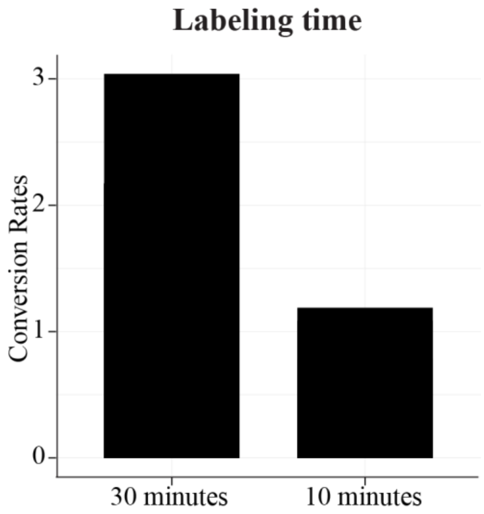

Supplementary figure 2. Labeling of the nascent transcription with 4-thiouridine (4sU) is directly proportional to the labeling time. Bar-plot depicts the conversion rates (T>C mutations) from SLAM-seq data generated after 30 minutes and 10 minutes of labeling with 4sU. The data for 30 minutes time-point in 22RV1 cell line was previously reported (GSE156884) and the data for 10 minutes time-point was generated in this study (22RV1 cell line).

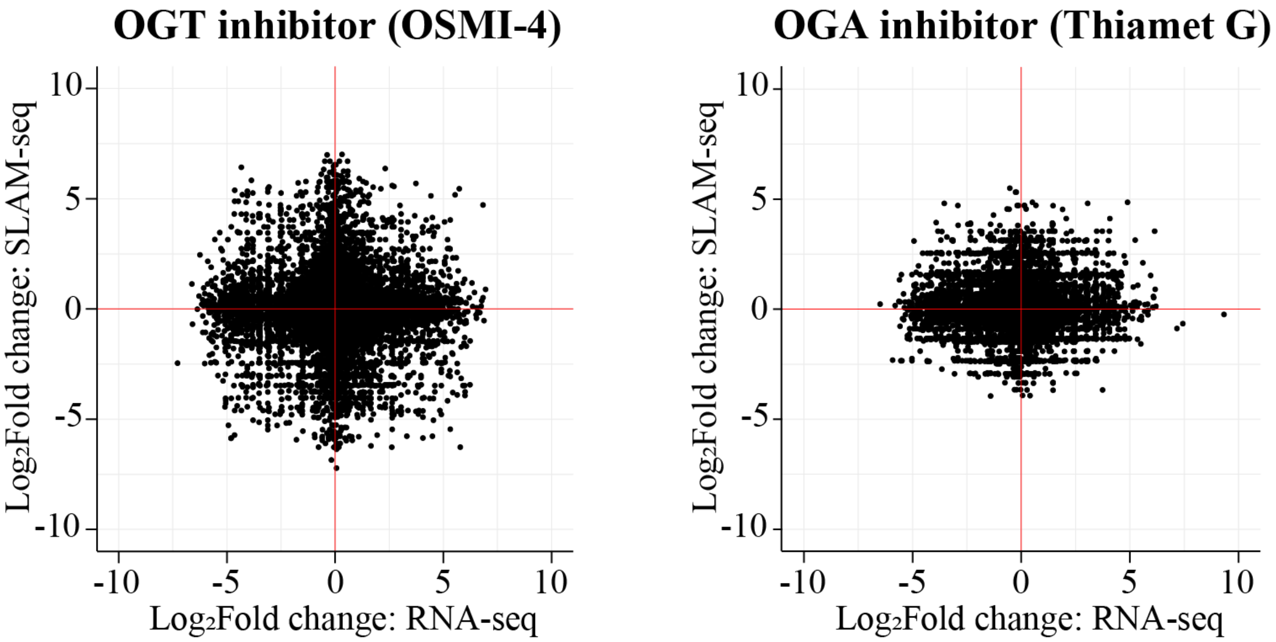

Supplementary figure 3. Correlation between SLAM-seq and RNA-seq data. We analyzed our SLAM-seq data with the SLAM-DUNK step to generate nascent mRNA profiles and without the SLAM-DUNK step to generate overall transcriptional profiles.

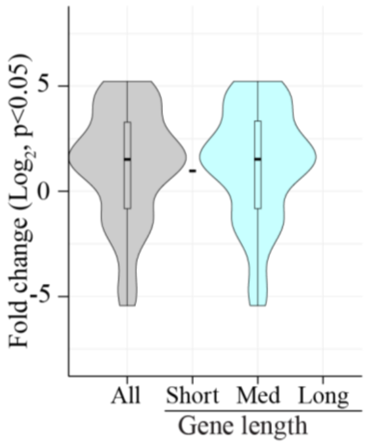

Supplementary figure 4. OGT inhibition does not result in gene-length dependent transcriptional defects. Violin plots representing the distribution of gene lengths for differentially expressed genes (DEGs) in OGT-inhibitor-treated SLAM-seq data. The distribution shows no significant difference in the nascent transcription according to gene length.

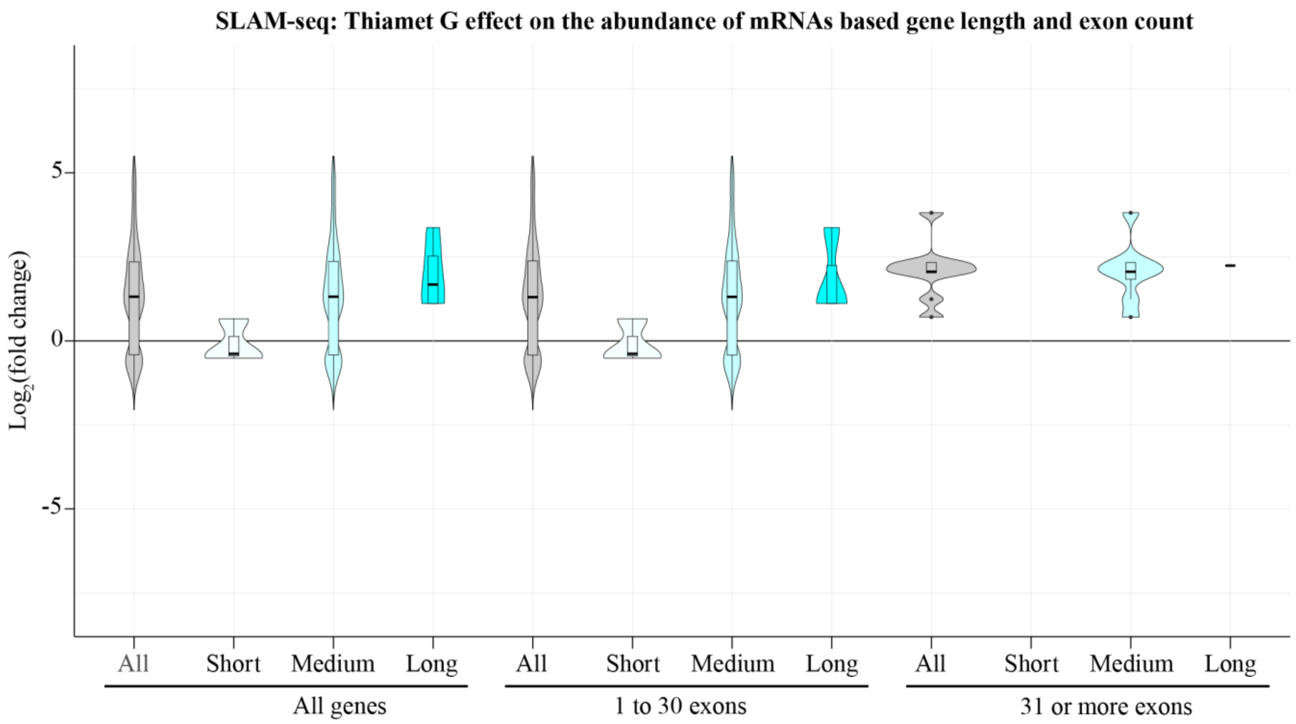

Supplementary figure 5. OGA inhibitor effect on nascent transcription according to gene length and exon count. The transcriptome was trichotomized according to gene-length and further subset according to exon count.

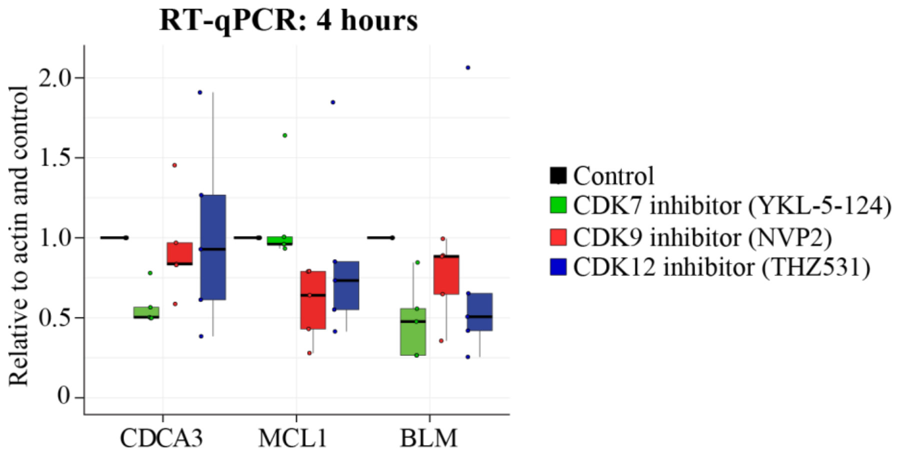

Supplementary figure 6. Validation of the target-selective effects of inhibitors targeting the transcriptional kinases. 22RV1 cells were treated with the inhibitors of transcriptional kinases (500 nM YKL-5-124, 20 nM NVP2 and 500 nM THZ531) for four hours followed by RNA isolation. CDCA3 is regulated by CDK7, MCL1 is target of CDK9 and BLM is regulated by CDK12. Data is from five biological replicates.

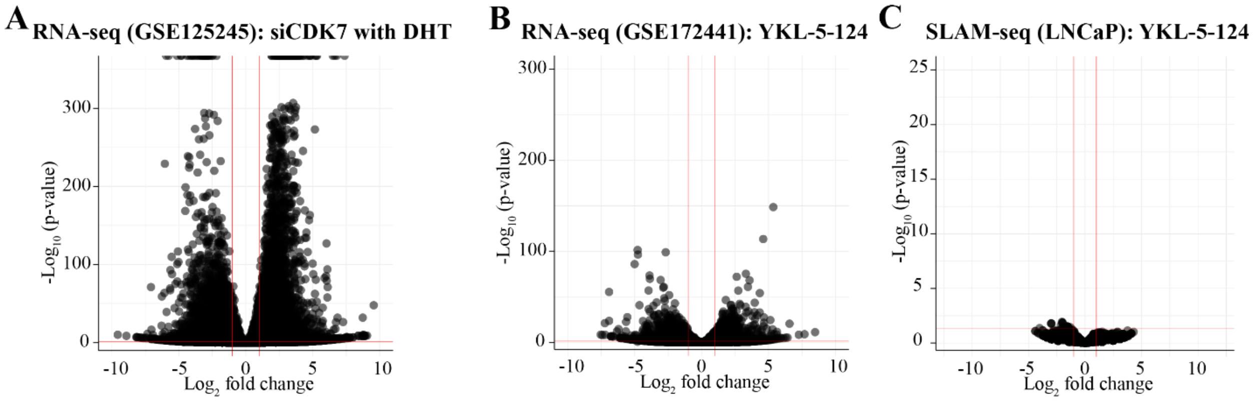

Supplementary figure 7. CDK7 inhibition affects transcriptional program but does not affect nascent transcription. A) Volcano plot of the DEGs in response to CDK7 knockdown performed in LNCaP cell line (analysis of previously generated data from GSE125245). DHT: 10 nM dihydrotestosterone. **B)** Volcano plot represents the differentially expressed genes (DEGs) in response to CDK7 inhibitor 100 nM YKL-5-1-24 (analysis of previously generated data from GSE172441, peripheral blood mononuclear cells treated with YKL-5-1-24 for 6 hours). **C)** Volcano plot of the nascent mRNAs from LNCaP cells treated with 500 nM YKL-5-124 for 4 hours (this manuscript). Samples were processed using SLAM-seq protocol and analyzed using SLAM-DUNK.

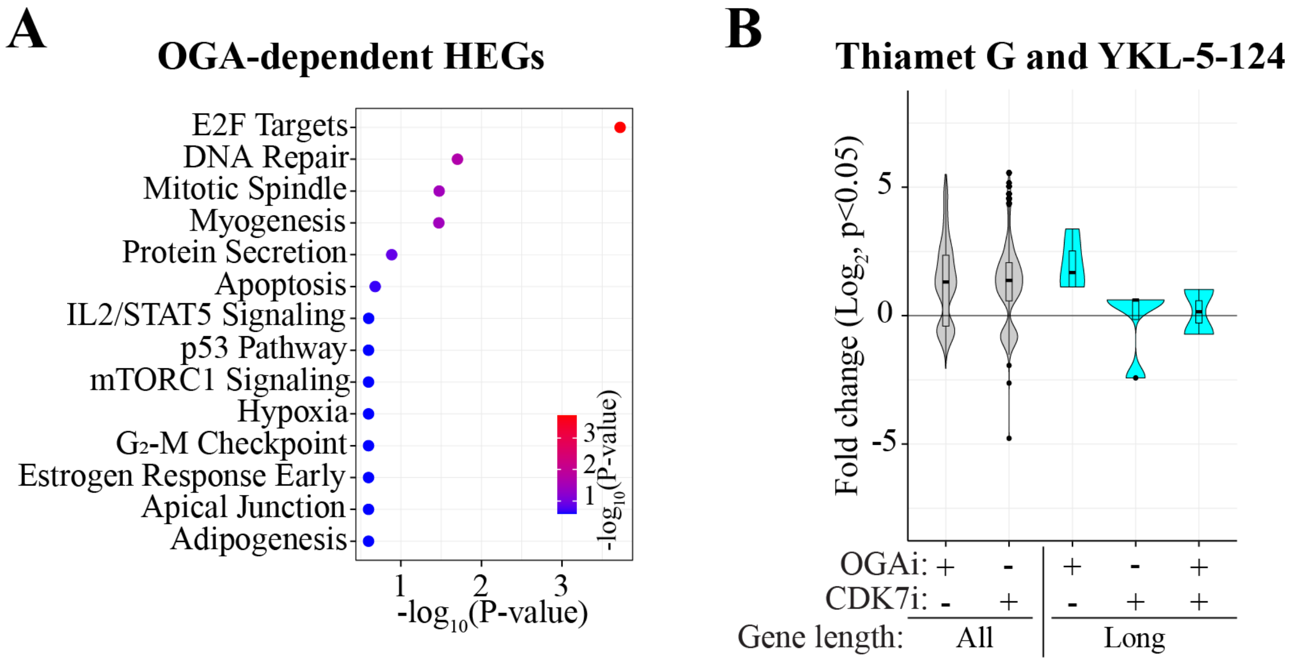

Supplementary figure 8. OGA stimulates transcription of E2F target genes. A) Gene set enrichment analysis of the OGA-dependent genes having more than 25 exons. These genes were defined as "High exon genes (HEGs)". **B)** CDK7 inhibition (500 nM YKL-5-124) prevents OGA inhibition-induced transcription of long genes (22RV1 SLAM-seq data).

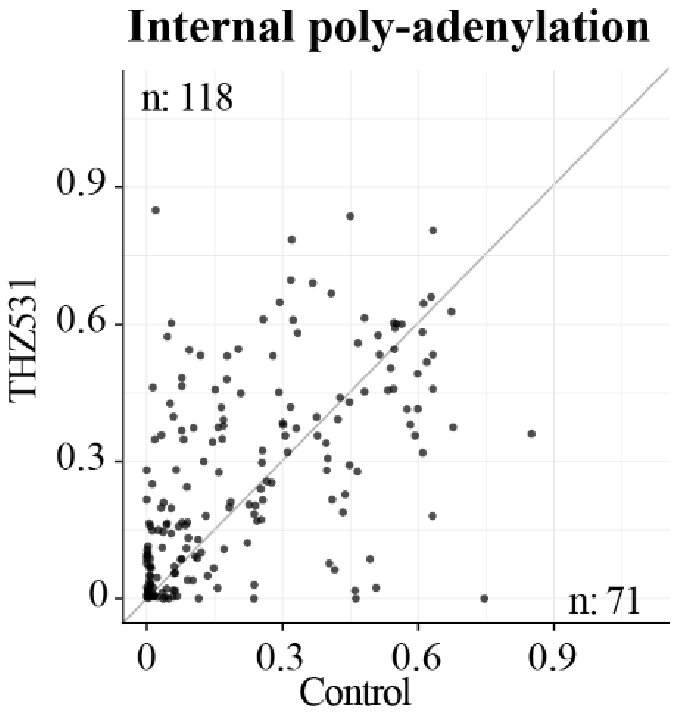

Supplementary figure 9. Inhibition of CDK12/13 induces intronic polyadenylation in prostate cancer cells. Intronic poly-adenylation sites (IPAs) were identified from the SLAM-seq data using IPAFinder. IPAFinder assigns an intronic poly(A) site usage index (IPUI) to putative IPAs. Scatter plot represents the IPUI from the control and CDK12/13 (THZ531) inhibitor treated samples. The number of IPA events identified in each condition are highlighted in the scatter plot.

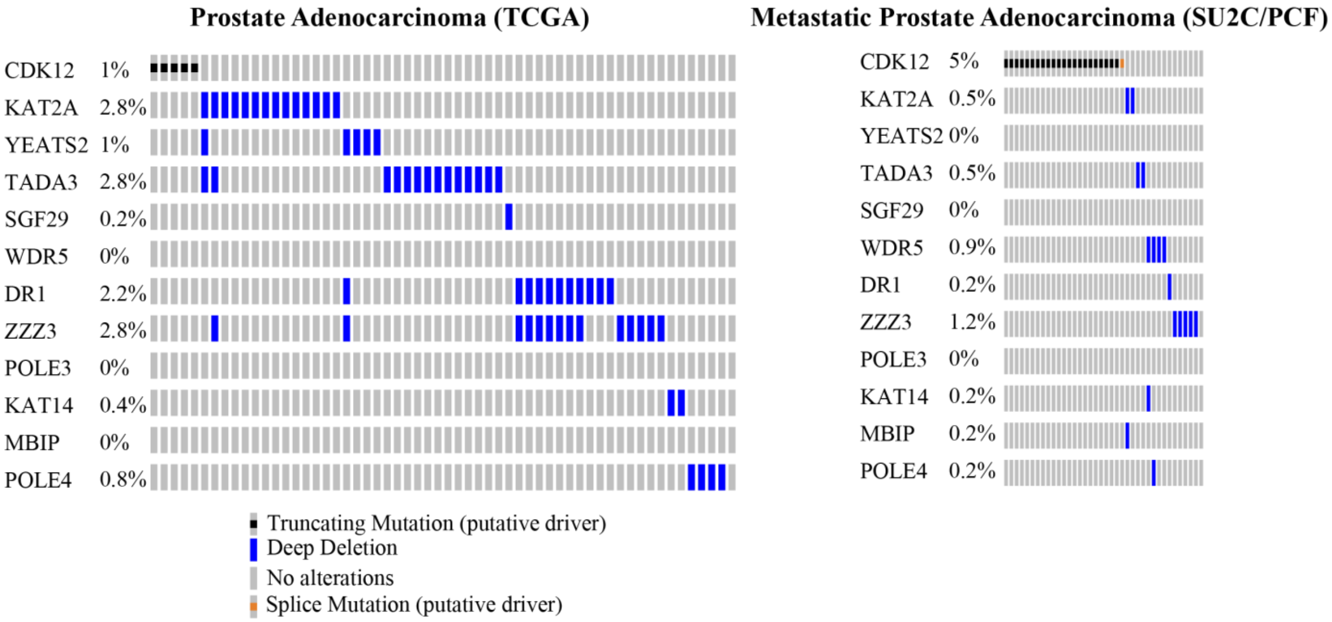

Supplementary figure 10. Inactivation of *CDK12* and mutations in ATAC complex proteins are mutually exclusive in prostate cancer patient samples. Prostate cancer (TCGA) and metastatic prostate cancer patient data (SU2C/PCF) accessed through the cBioportal were used to identify the patients having *CDK12* truncating mutations and homozygous deletion in any of the ATAC complex genes. Both the mutation signatures are found in a small subset of data and show mutual exclusivity. The percentage indicates the occurrence of the mutational event in the entire sampled patient data.

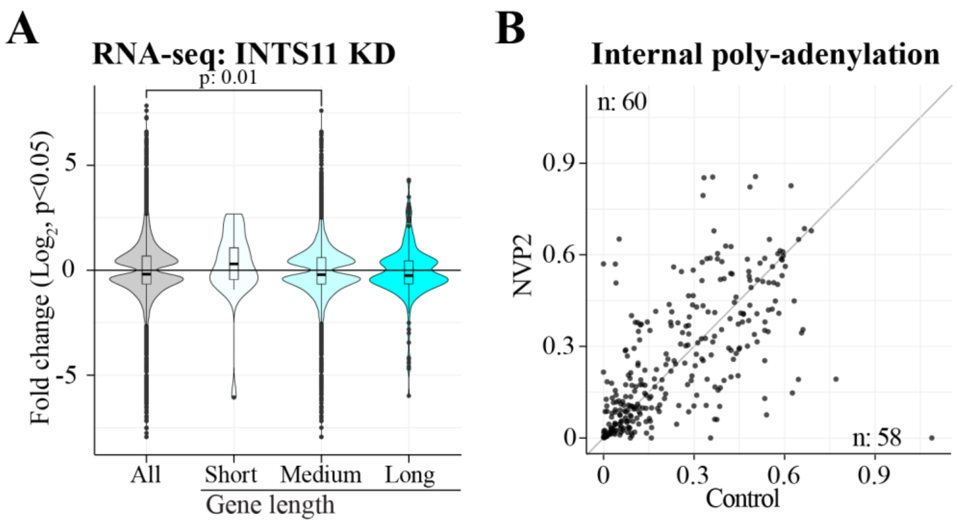

Supplementary figure 11. Validation of CDK9 inhibitor effects. A) The loss of the integrator complex proteins stimulates the transcription of short genes, mimicking the effects of the CDK9 inhibition. To evaluate the effects, we used the gene-expression table deposited on GEO to call the DEGs ($p < 0.05$ and $log_2FC \pm 1$) from the INST11 knockdown RNA-seq data (GSE125534) and evaluated the impact on DEGs according to gene-length. Students t-test was used to calculate the p-value. **B)** Intronic poly-adenylation sites (IPAs) were identified from the SLAM-seq data using IPAFinder. IPAFinder assigns an intronic poly(A) site usage index (IPUI) to putative IPAs. Scatter plot represents the IPUI from the control and CDK9 (NVP2) inhibitor treated samples. The number of IPA events identified in each condition are highlighted in the scatter plot.

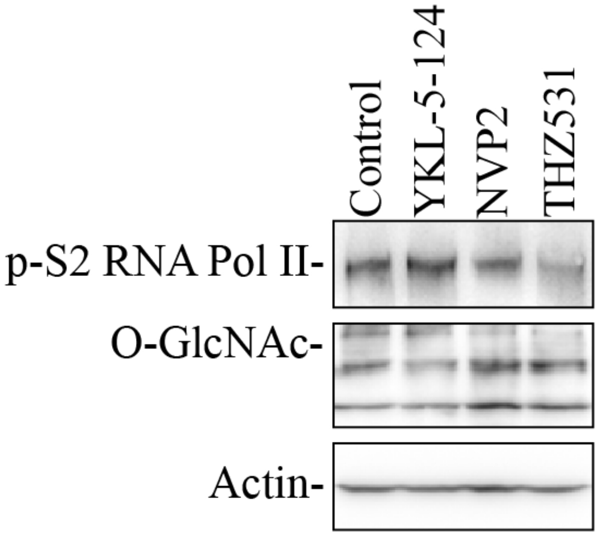

Supplementary figure 12. Inhibition of CDK9 and CDK12/13 increase overall O-GlcNAcylation. 22RV1 cells were treated as indicated for 4 hours and samples analyzed using western blot. Doses used: 500 nM YKL-5-124, 20 nM NVP2, 500 nM THZ531.

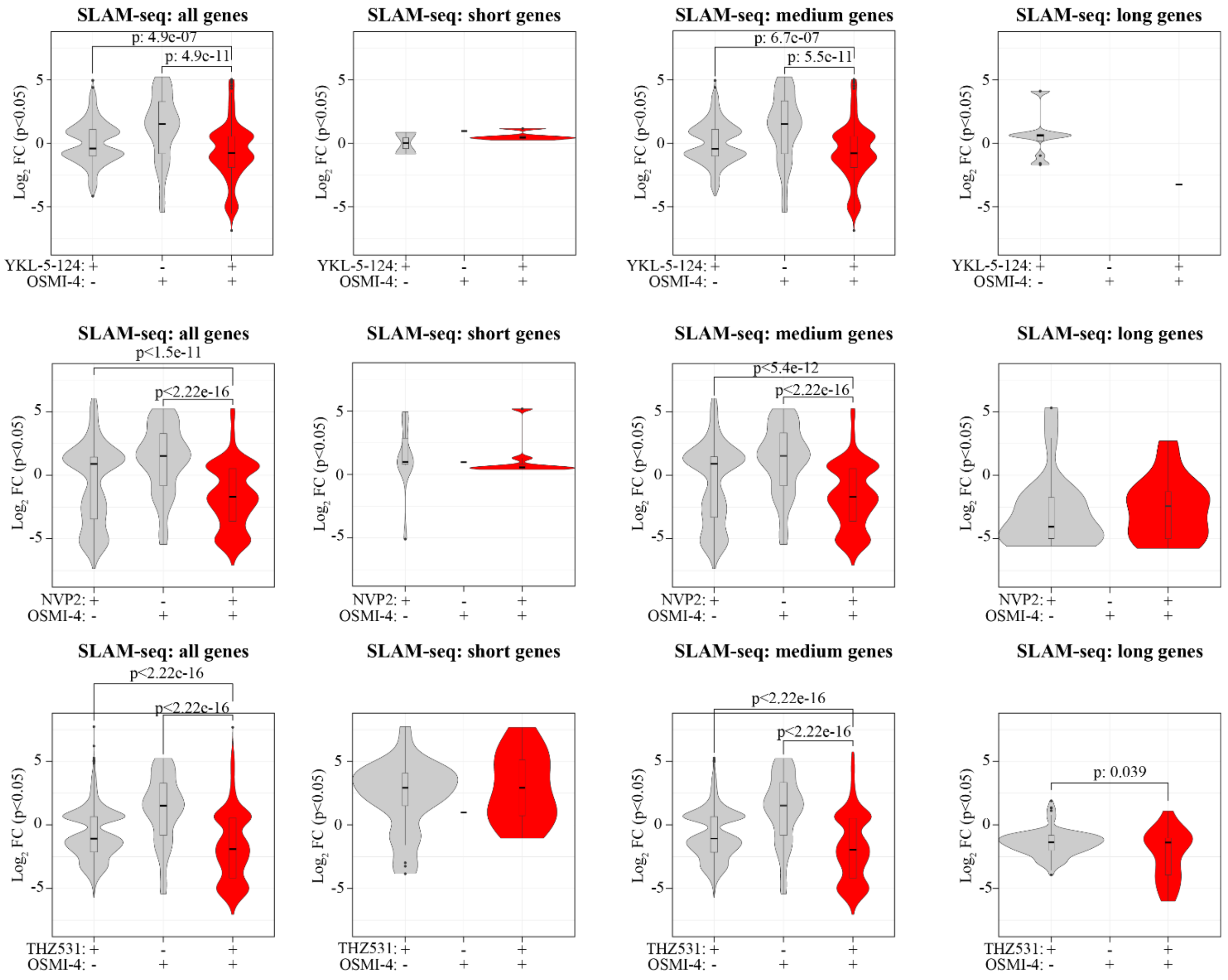

Supplementary figure 13. Inhibition of OGT augments the CDK9- and CDK12-inhibitor effects on nascent transcriptome. Cells were treated for 4 hours, samples processed using SLAM-seq protocol and analyzed using SLAM-DUNK. Doses used: 500 nM YKL-5-124, 20 nM NVP2, 500 nM THZ531, 20 µM OSMI-4. Students t-test was used to calculate the p-value.

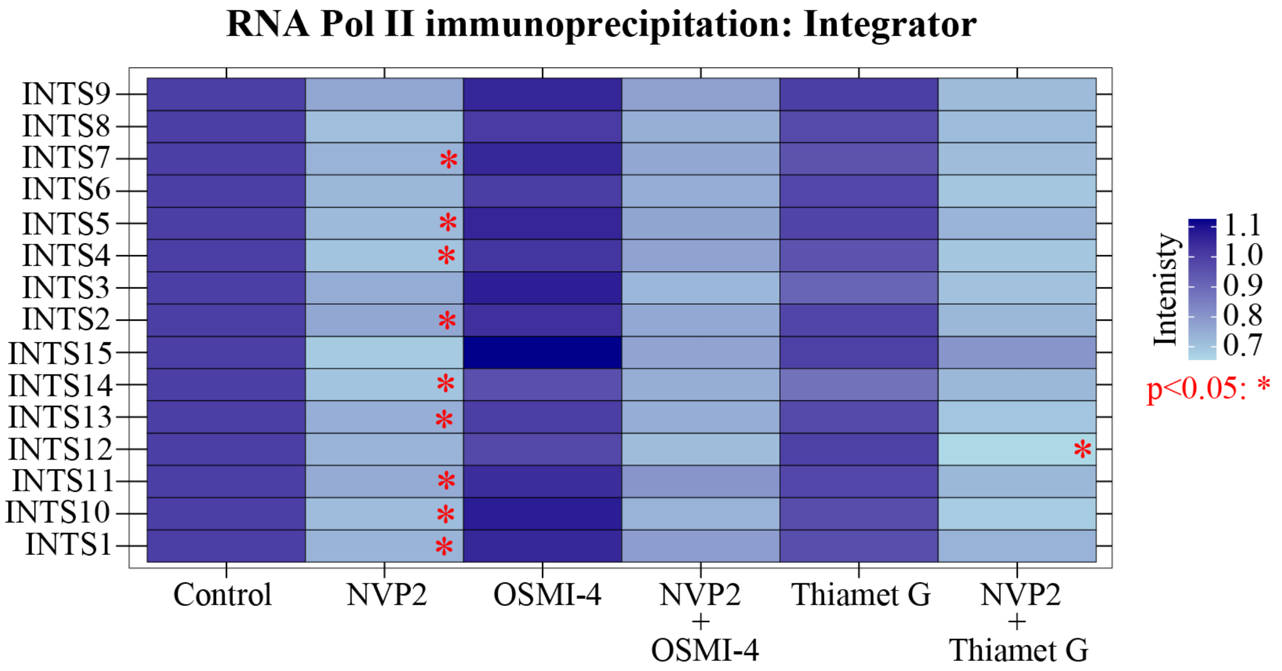

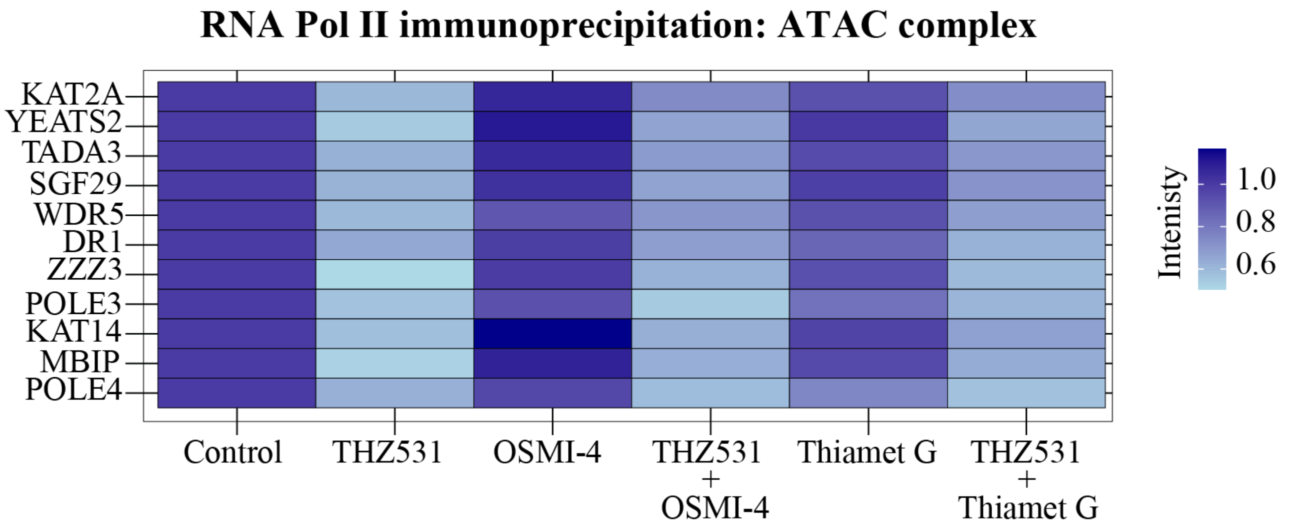

Supplementary figure 14. Loss of the integrator complex and the ATAC complex in response to CDK9 and CDK12/13 inhibition, respectively, is independent of dynamic O-GlcNAcylation. The RNA Pol II-normalized intensity values of the Integrator complex proteins in response to the indicated treatments presented relative to the untreated condition. The RNA Pol II-normalized intensity values of the ATAC complex proteins in response to the mentioned treatments presented relative to the untreated condition.

Cell line: 22RV1

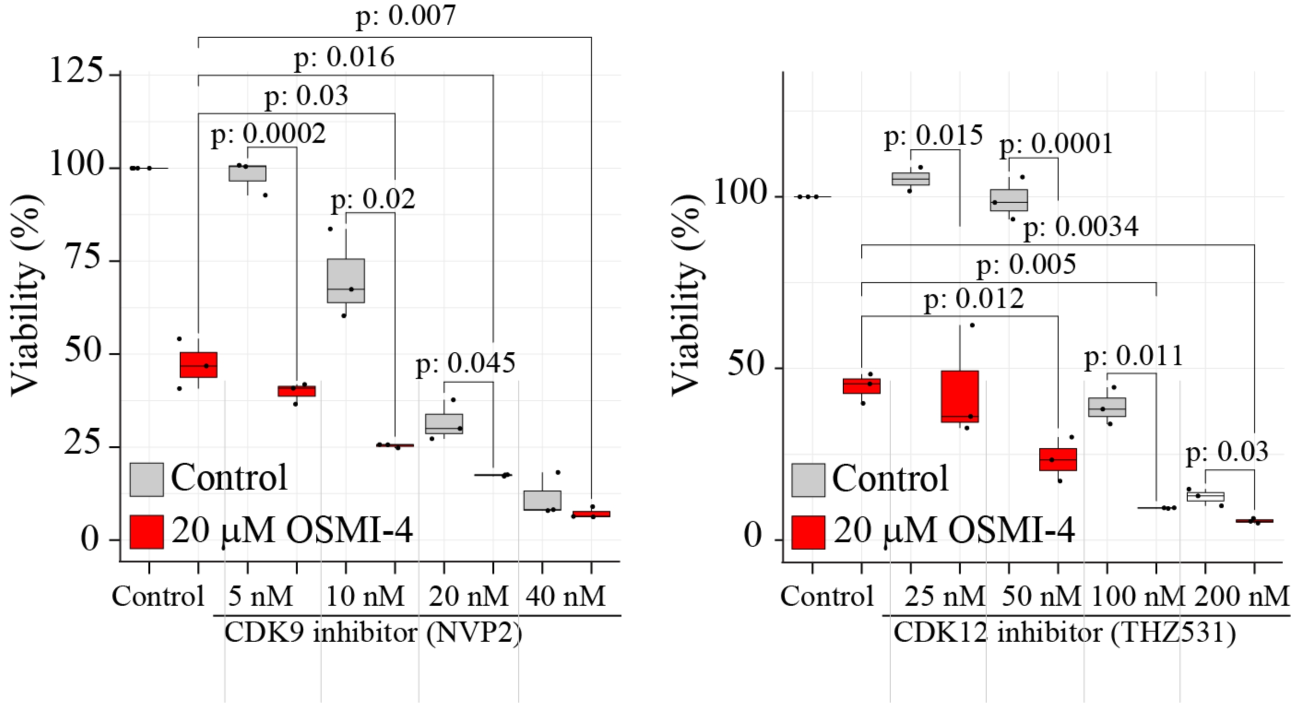

Cell line: C4-2

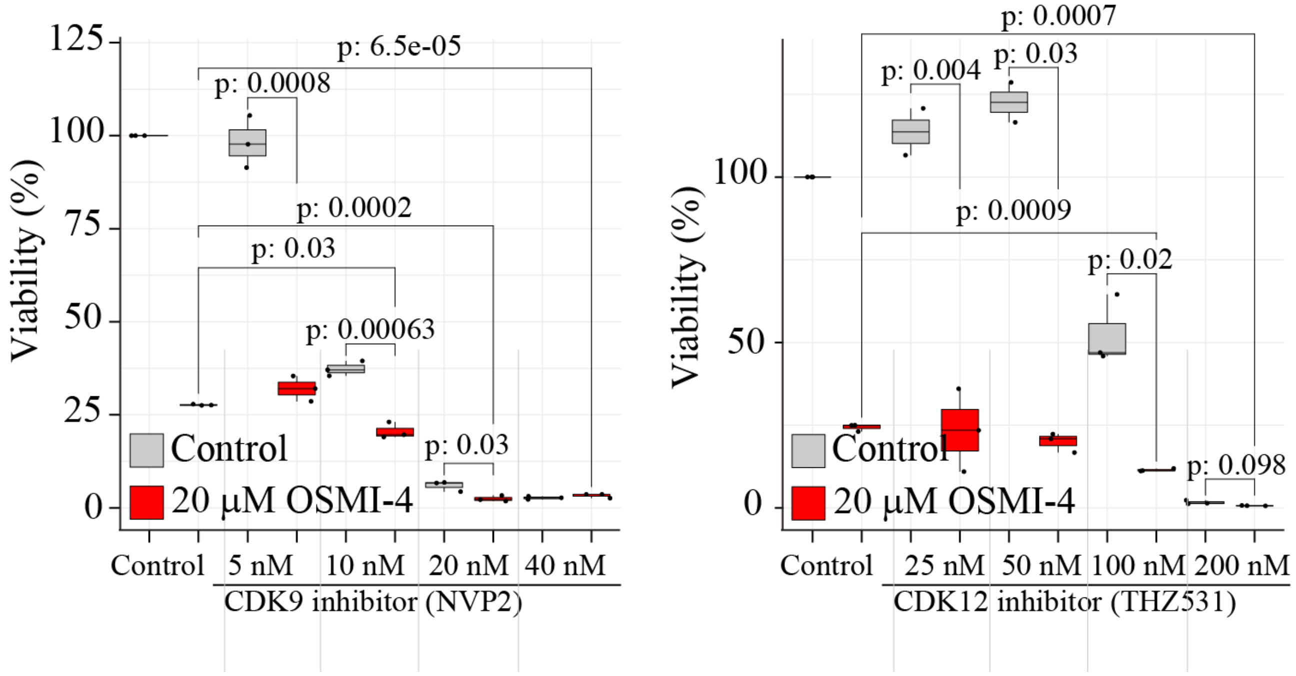

Supplementary figure 15. OGT inhibition augments the anti-proliferative effects of CDK9 and CDK12/13 inhibitors. 22RV1 and C4-2 cells were treated with increasing doses of CDK9 and CDK12/13 inhibitors in combination with 20 μM OSMI-4. Cell viability was measured using CellTiterGlo (n= 3 biological replicates each having 3 technical replicates). Statistical significance was calculated using the Students t-test.

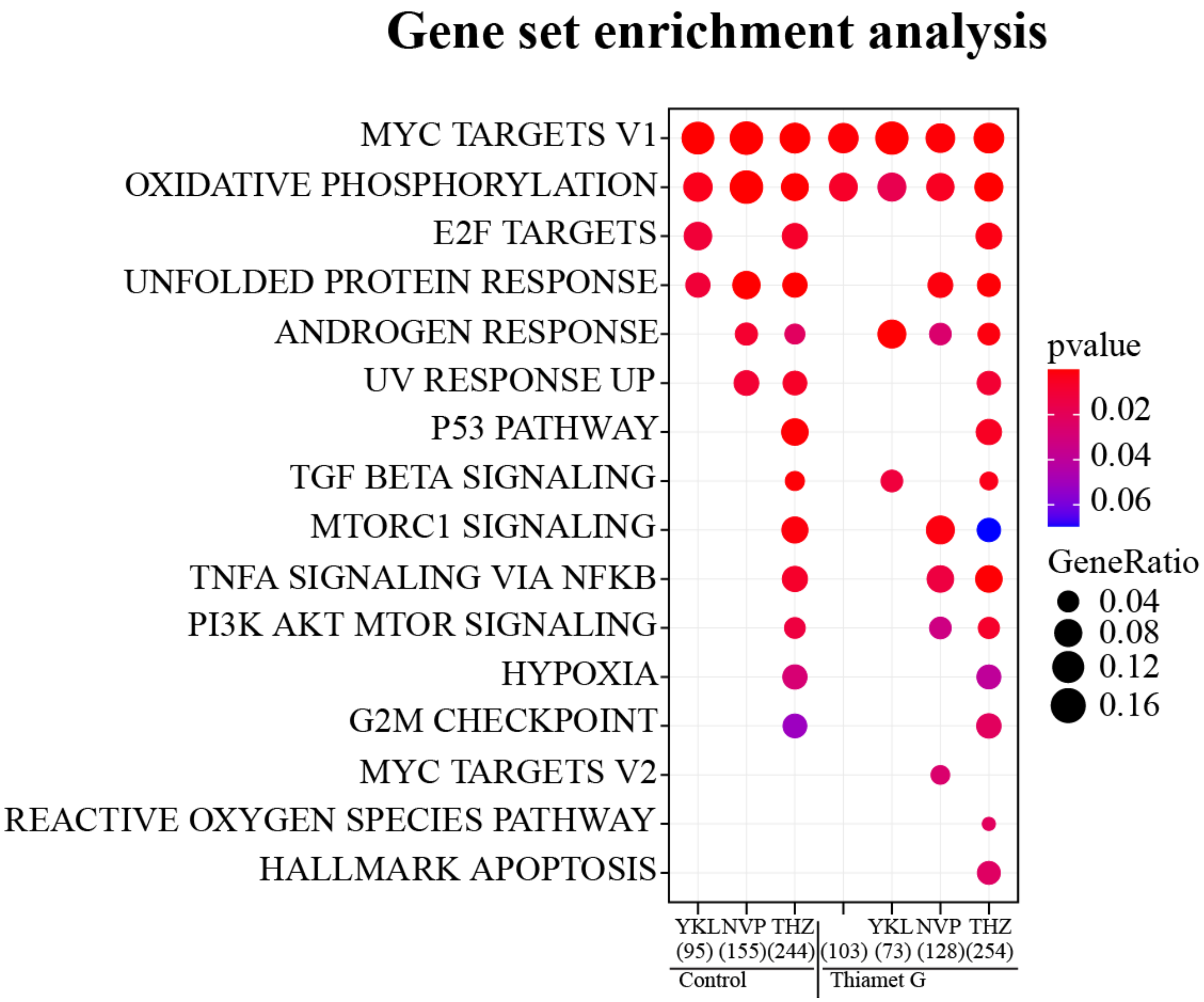

Supplementary figure 16. Depleting OGA activity does not affect the transcriptional program induced by inhibitors of CDK9 or CDK12/13. Gene set enrichment analysis of the SLAM-seq data generated with the transcriptional kinase inhibitors either in the presence or absence of the OGA inhibitor.

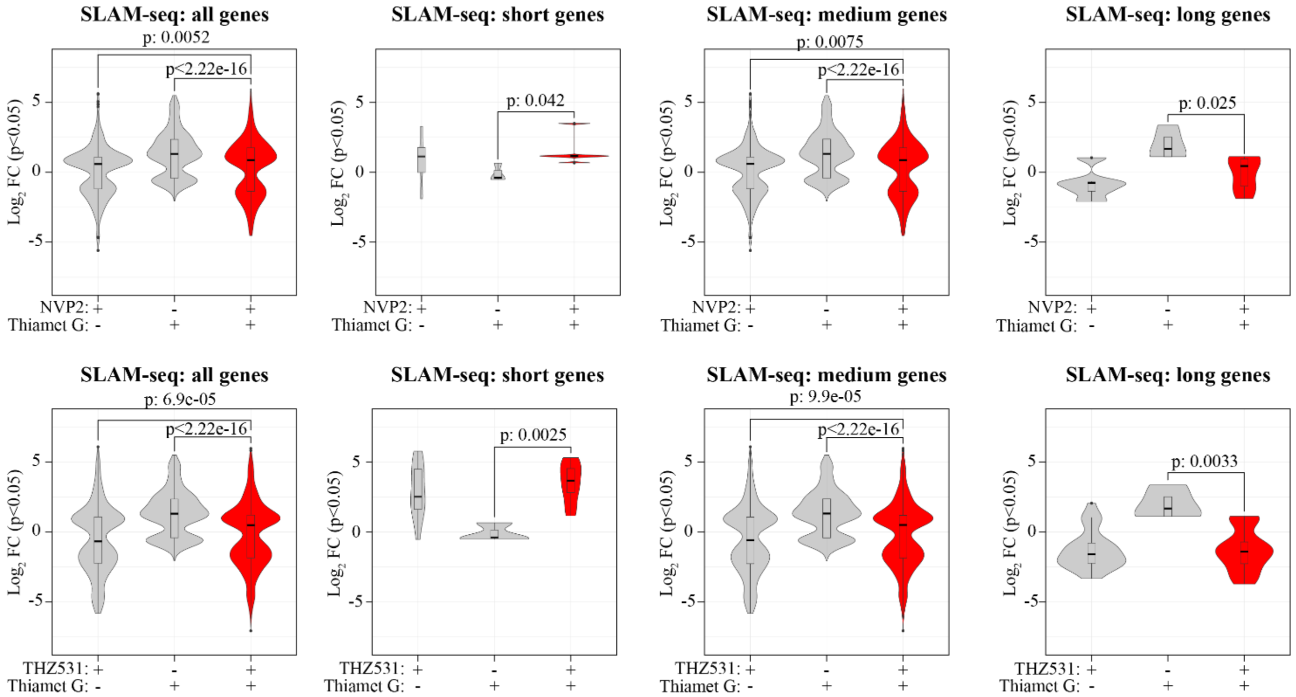

Supplementary figure 17. Depletion of OGA activity rescues CDK9- and CDK12/13-inhibitor effects on mRNA maturation. Cells were treated for 4 hours, samples processed using SLAM-seq protocol and analyzed using SLAM-DUNK. Doses used: 20 nM NVP2, 500 nM THZ531 and 20 μM Thiamet G. Students t-test was used to calculate the p-value.

Cell line: 22RV1

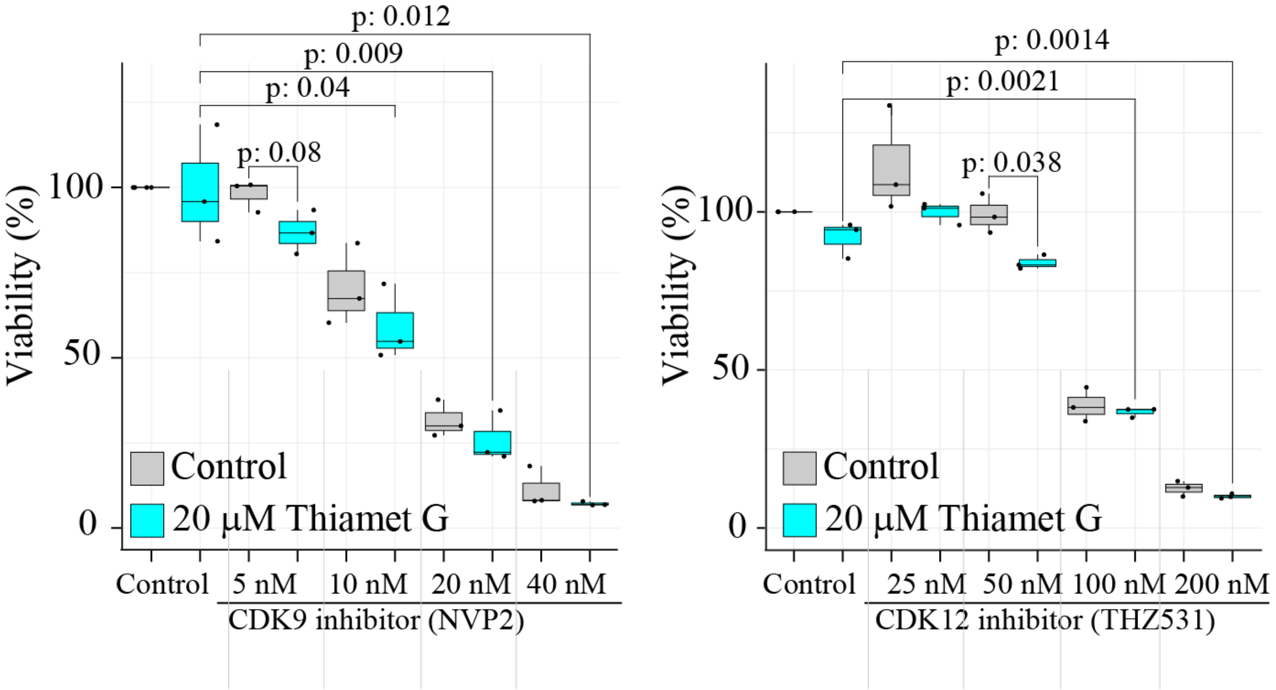

Cell line: C4-2

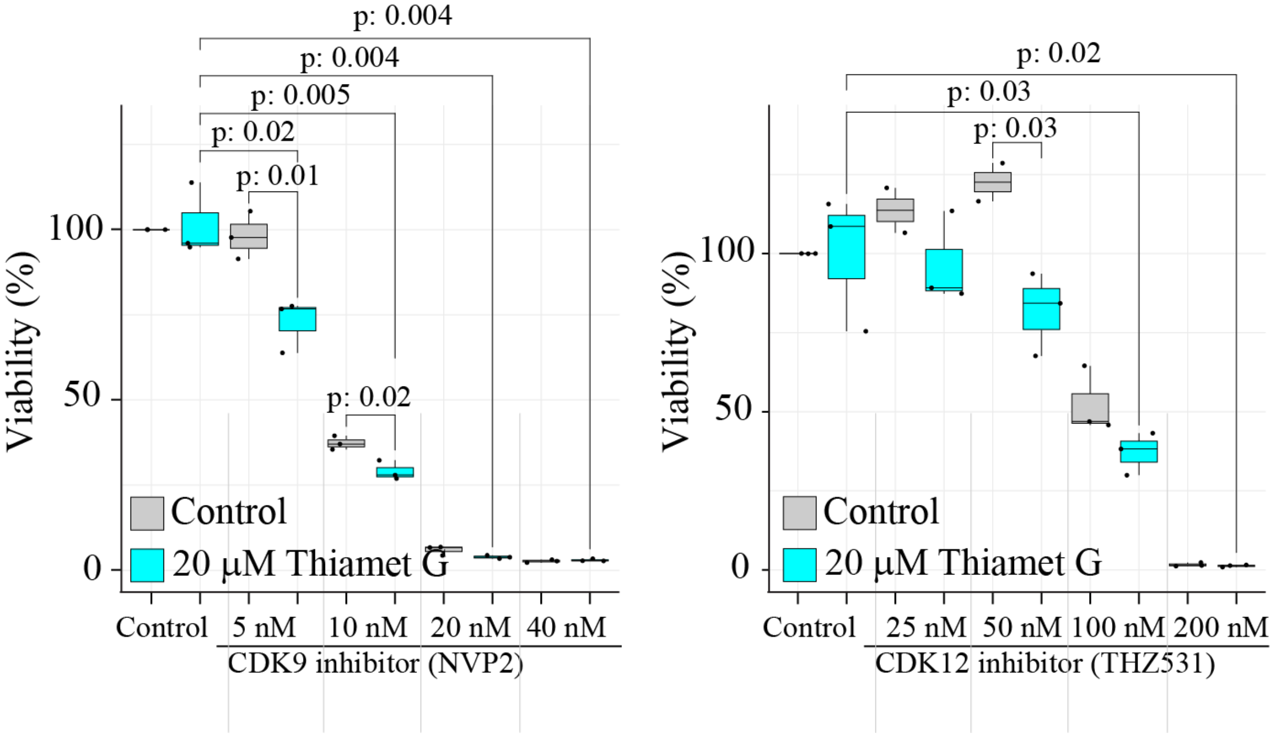

Supplementary figure 18. OGA inhibition augments the anti-proliferative effects of CDK9 and CDK12/13 inhibitors. 22RV1 and C4-2 cells were treated with increasing doses of CDK9 and CDK12/13 inhibitors in combination with 20 µM Thiamet G. Cell viability was measured using CellTiterGlo (n= 3 biological replicates each having 3 technical replicates). Statistical significance was calculated using the Students t-test.

Supplementary table 1. 22RV1 cells were simultaneously treated with the transcriptional kinase inhibitors or 20 µM OSMI-4 / Thiamet G for 4 hours, followed by RNA Pol II immunoprecipitation. These samples were analyzed using mass spectrometry. The raw intensity values were first normalized to RNA Pol II and the fold change was calculated relative to untreated sample. Students t-test was used to calculate the significance from three biological replicates. The significantly gained or lost proteins from RNA Pol II were subjected to enrichment using String database. The 'Summary' sheet has the detailed description of the subsequent sheets.

Supplementary Table 2. Detailed description of the reagents and the cell line models used in this study.
