# Supplementary material for: Dynamic O-GlcNAcylation and phosphorylation attract and expel proteins from RNA polymerase II to regulate mRNA maturation
Source: J Biomed Sci. 2025 Apr 4;32:39. doi: 10.1186/s12929-025-01135-9 (PMC11969731; doi:10.1186/s12929-025-01135-9)
Supplement: Supplementary file 3 — Supplementary material 3. [file 12929_2025_1135_MOESM3_ESM.pdf]

Supplementary Table 2. Detailed description of the reagents and the cell line models used in this study.

Reagent	Manufacturer	Catalog number
Antibodies		
Total Pol II	Santa Cruz Biotechnology	sc-56767
Total Pol II	Santa Cruz Biotechnology	sc-47701
Pol II p-Ser 2	Cell Signalling Technologies	CST-13499
Pol II p-Ser 5	Cell Signalling Technologies	CST-13523
Acetyl-Histone H3 (Lys9)	Cell Signalling Technologies	CST-9649
Histone H3 (D1H2) XP® Rabbit mAb	Cell Signalling Technologies	CST-4499
Mouse monoclonal: RL2	Abcam	ab2739
Actin	Abcam	ab49900
Inhibitors and other reagents		
YKL-5-124 (CDK7 inhibitor)	MedChemExpress	HY-101257
NVP2 (CDK9 inhibitor)	MedChemExpress	HY-12214A
THZ531 (CDK12 inhibitor)	MedChemExpress	HY-103618
OSMI-4 (OGT inhibitor)	MedChemExpress	HY-114361
Thiamet-G (OGA inhibitor)	MedChemExpress	HY-12588
Protein A/G Magnetic Beads	MedChemExpress	HY-K0202
Protease inhibitor	MERCK	535142-1ML
Phosphatase inhibitor	MERCK	524633-1ML
4-thiouridine	ThermoFisher Scientific	215213405
Iodoacetamide	ThermoFisher Scientific	11467736
Immobilon-E transfer membrane	ThermoFisher Scientific	16379591
Commercial kits		
RNAspin mini kit	Cytiva	25050071
QuantSeq 3' mRNA-Seq library preparation kit (FWD)	Lexogen	
qScript cDNA Synthesis Kit	QuantaBio	95047-100
Deposited data		
SLAM-seq (22RV1)	Sequence Read Archive (SRA)	PRJNA1108610
SLAM-seq (LNCaP)	Sequence Read Archive (SRA)	PRJNA1118101
Experimental models: Cell lines		
Human prostate cancer: 22RV1	ATCC	
Human prostate cancer: LNCaP	ATCC	
Human prostate cancer: C4-2	ATCC	
Primer sequences		
CTGGAGGGTCTTAAACATGCC	Integrated DNA Technologies	CDCA3 For
CACTGCTGGTCTTCATAGGTG	Integrated DNA Technologies	CDCA3 Rev
TGCTTCGGAAACTGGACATCA	Integrated DNA Technologies	MCL1 For
TAGCCACAAAGGCACCAAAAG	Integrated DNA Technologies	MCL1 Rev
GGACCTTGACACCTCTGACAG	Integrated DNA Technologies	BLM For
GGATTCAGCTCCTGCATACTCA	Integrated DNA Technologies	BLM Rev
TGGGACGACATGGAGAAAAT	Integrated DNA Technologies	ACTIN For
AGAGGCGTACAGGGATAGCA	Integrated DNA Technologies	ACTIN Rev

Computational tools		
FastQC		https://www.bioinformatics.babraham.ac.uk/projects/fastqc/
Bowtie 2 Aligner		https://bowtie-bio.sourceforge.net/bowtie2/manual.shtml#obtaining-bowtie-2
STAR Aligner		https://github.com/alexdobin/STAR
Samtools		https://www.htslib.org/
Deeptools		https://github.com/deeptools/deepTools
IPAFinder		https://github.com/ZhaozzReal/IPAFinder
SLAM-DUNK		https://t-neumann.github.io/slamdunk/
